# The Proteomic Code: a molecular recognition code for proteins

**DOI:** 10.1186/1742-4682-4-45

**Published:** 2007-11-13

**Authors:** Jan C Biro

**Affiliations:** 1 Homulus Foundation, 88 Howard, #1205, San Francisco, CA 94105, USA

## Abstract

**Background:**

The Proteomic Code is a set of rules by which information in genetic material is transferred into the physico-chemical properties of amino acids. It determines how individual amino acids interact with each other during folding and in specific protein-protein interactions. The Proteomic Code is part of the redundant Genetic Code.

**Review:**

The 25-year-old history of this concept is reviewed from the first independent suggestions by Biro and Mekler, through the works of Blalock, Root-Bernstein, Siemion, Miller and others, followed by the discovery of a Common Periodic Table of Codons and Nucleic Acids in 2003 and culminating in the recent conceptualization of partial complementary coding of interacting amino acids as well as the theory of the nucleic acid-assisted protein folding.

**Methods and conclusions:**

A novel cloning method for the design and production of specific, high-affinity-reacting proteins (SHARP) is presented. This method is based on the concept of proteomic codes and is suitable for large-scale, industrial production of specifically interacting peptides.

## Background

Nucleic acids and proteins are the carriers of most (if not all) biological information. This information is complex, well organized in space and time. These two kinds of macromolecules have polymer structures. Nucleic acids are built from four nucleotides and proteins are built from 20 amino acids (as basic units). Both nucleic acids and proteins can interact with each other and in many cases these interactions are extremely strong (*K*_d _~ 10^-9^-10^-12 ^M) and extremely specific. The nature and origin of this specificity is well understood in the case of nucleic acid-nucleic acid (NA-NA) interactions (DNA-DNA, DNA-RNA, RNA-RNA), as is the complementarity of the Watson-Crick (W-C) base pairs. The specificity of NA-NA interactions is undoubtedly determined at the basic unit level where the individual bases have a prominent role.

**Figure 1:**
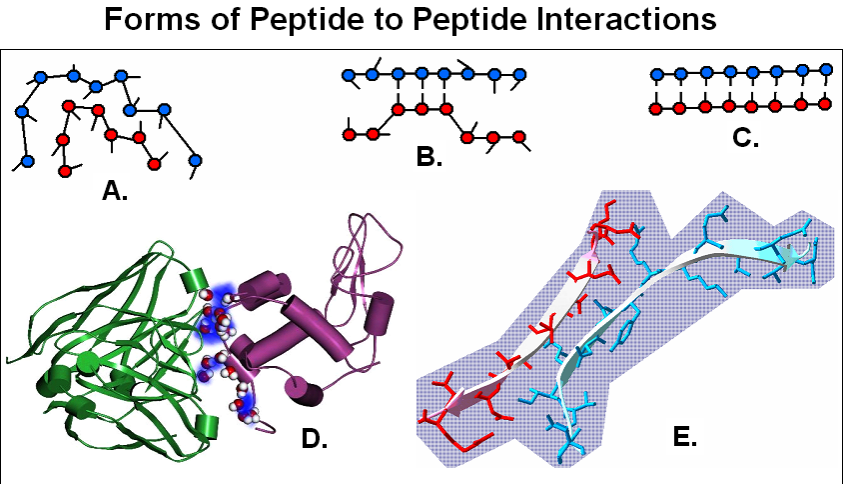
Forms of peptide to peptide interactions. The specificity of interactions between two peptides might be explained in two ways. First, many amino acids collectively form larger configurations (protrusions and cavities, charge and hydropathy fields) which fit each other (A and D). Second, the physico-chemical properties (size, charge, hydropathy) of individual amino acids fit each other like "lock and key" (C and E). There are even intermediate forms (B).

Our most established view on the specificity of protein-protein (P-P) interactions is completely different [1]. In this case the amino acids in a particular protein together establish a large 3D structure. This structure has protrusions and cavities, charged and uncharged areas, hydrophobic and hydrophilic patches on its surface, which altogether form a complex 3D pattern of spatial and physico-chemical properties. Two proteins will specifically interact with each other if their complex 3D patterns of spatial and physico-chemical properties fit to each other as a mold to its template or a key to its lock. In this way the specificity of P-P interactions is determined at a level higher than the single amino acid (Figure 1).

The nature of specific nucleic acid-protein (NA-P) interactions is less understood. It is suggested that some groups of bases together form 3D structures that fits to the 3D structure of a protein (in the case of single-stranded nucleic acids). Alternatively, a double-stranded nucleic acid provides a pattern of atoms in the grooves of the double strands, which is in some way specifically recognized by nucleo-proteins [2].

Regulatory proteins are known to recognize specific DNA sequences directly through atomic contacts between protein and DNA, and/or indirectly through the conformational properties of the DNA.

There has been ongoing intellectual effort for the last 30 years to explain the nature of specific P-P interactions at the residue unit (individual amino acid) level. This view states that there are individual amino acids that preferentially co-locate in specific P-P contacts and form amino acid pairs that are physico-chemically more compatible than any other amino acid pairs. These physico-chemically highly compatible amino acid pairs are complementary to each other, by analogy to W-C base pair complementarity.

The comprehensive rules describing the origin and nature of amino acid complementarity is called the Proteomic Code.

## The history of the Proteomic Code

### People from the past

This is a very subjective selection of scientists for whom I have great respect; I believe they contributed – in one way or another – to the development of the Proteomic Code.

**Linus Pauling **is regarded as "the greatest chemist who ever lived". *The Nature of the Chemical bond *is fundamental to the understanding of any biological interaction [3]. His works on protein structure are classics [4]. His unconfirmed DNA model, in contrast to the established model, gives some theoretical ideas on how specific nucleic acid-protein interactions might happen [5,6].

**Carl R Woese **is famous for defining the Archaea, the third life form on Earth (in addition to bacteria and eucarya). He also proposed the "RNA world" hypothesis. This theory proposes that a world filled with RNA (ribonucleic acid)-based life predates current DNA (deoxyribonucleic acid)-based life. RNA, which can store information like DNA *and *catalyze reactions like proteins (enzymes), may have supported cellular or pre-cellular life. Some theories about the origin of life present RNA-based catalysis and information storage as the first step in the evolution of cellular life.

The RNA world is proposed to have evolved into the DNA and protein world of today. DNA, through its greater chemical stability, took over the role of data storage while proteins, which are more flexible as catalysis through the great variety of amino acids, became the specialized catalytic molecules. The RNA world hypothesis suggests that messenger RNA (mRNA), the intermediate in protein production from a DNA sequence, is the evolutionary remnant of the "RNA world" [7].

Woese's concept of a common origin of our nucleic acid and protein "worlds" is entirely compatible with the foundation of the Proteomic Code.

**Margaret O Dayhoff **is the mother of bioinformatics. She was the first who collected and edited the *Atlas of Protein Sequence and Structure *[8] and later introduced statistical methods into protein sequence analyses. Her work was a huge asset and inspiration to my first suggestion of the Proteomic Code [9-11].

**Figure 2:**
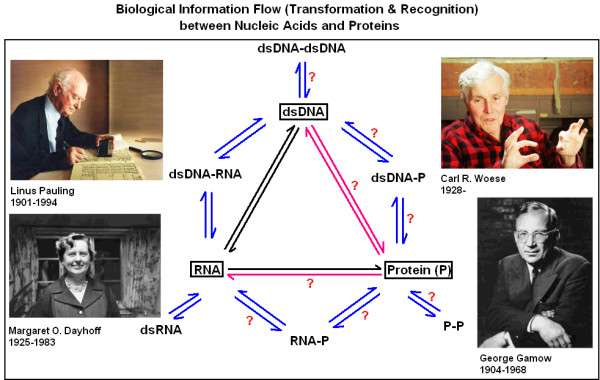
Biological information flow (transformation and recognition) between nucleic acids and proteins. All biological information is stored in nucleic acids (DNA/RNA) and much in proteins (P). The information transfer and interactions between nucleic acids and the formation of double-stranded (ds) forms are well known and understood. However, the exact nature of P-P and P-nucleic acid interactions is still obscure. The works of these four scientists played important roles in much that we know about such information transfers and interactions (subjectively chosen by the author of this article).

**George Gamow **was a theoretical physicist and cosmologist and spent only a few years in Cambridge, UK, but he was there when the structure of DNA was discovered in 1953. He developed the first genetic code, which was not only an elegant solution for the problem of information transfer from DNA to proteins, but at the same time explained how DNA might specifically interact with proteins [12-17]. In his mind, the codons were mirror images of the coded amino acids and they had very intimate relationships with each other. His genetic code proved to be wrong and the nature of specific nucleic acid-protein interactions is still not known, but he remains a strong inspiration (Figure 2) [18,19].

## First generation models for the Proteomic Code

The first generation models (up to 2006) of the novel Proteomic Code are based on perfect codon complementarity coding of interacting amino acid pairs.

### Mekler

Mekler described an idea of sense and complementary peptides that may be able to interact specifically, mediated by specific through-space, pairwise interactions between amino acid residues [20]. He suggested that amino acids of specifically interacting proteins, in their specifically interacting domains, are composed of two parallel sequences of amino acid pairs that are spatially complementary to each other, similarly to the Watson-Crick base pairs in nucleic acids. The protein/nucleic acid analogy in his theory was sustained and he proposed that these spatially complementary amino acids are coded by reverse-complementary codons (translational reading in the 5'→3' direction).

It is possible to segregate 64 (the number of different codons, including the three stop codons) of all the possible putative amino acid pairs (20 × 20/2 = 200) into three non-overlapping groups [21].

### Biro

I was also inspired by the complementarity of nucleic acids and developed a theory of complementary coding of specifically interacting amino acids [9-11]. I had no knowledge of the publications of Mekler or Idlis (published in two Russian papers). I was also convinced that amino acid pairs coded by complementary codons (whether in the same 5'→3'/5'→3' or opposite 5'→3'/3'→5' orientations) are somehow special and suggested that these pairs of amino acids might be responsible for specific intra- and intermolecular peptide interactions.

**Figure 3:**
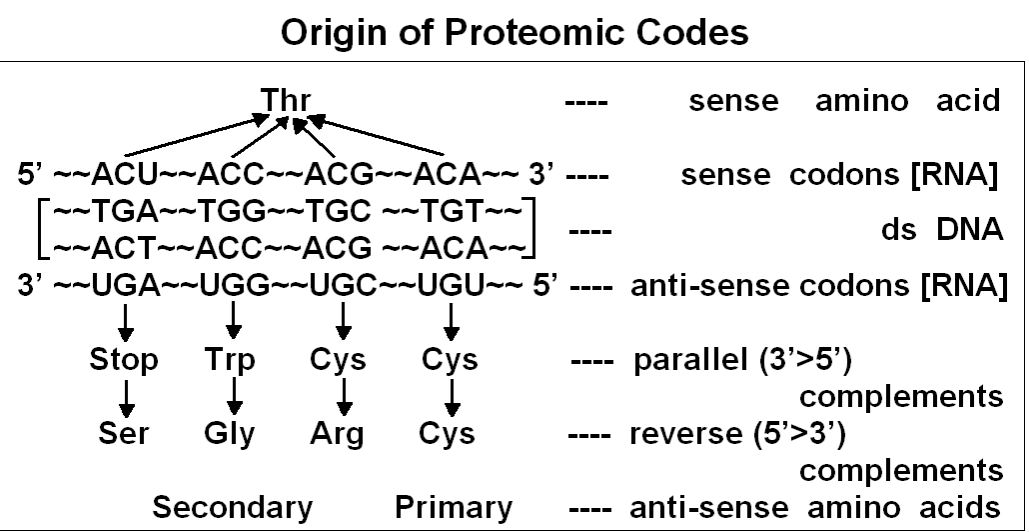
Origin of the Proteomic Code. Threonine (Thr) is coded by 4 different synonymous codons. Complementary triplets encode different amino acids in parallel (3'→5') and anti-parallel (5'→3') readings. Amino acids encoded by symmetrical codons are called "primary" and others "secondary" anti-sense amino acids (modified from [9].

I developed a method for pairwise computer searching of protein sequences for complementary amino acids and found that these specially coded amino acid pairs are statistically overrepresented in those proteins known to interact with each other. In addition, I was able to find short complementary amino acid sequences within the same protein sequences and inferred that these might play a role in the formation or stabilization of 3D protein structures (Figure 3). Molecular modeling showed the size compatibility of complementary amino acids and that they might form bridges 5–7 atoms long between the alpha C atoms of amino acids. It was a rather ambitious theory at a time when the antisense DNA sequences were called nonsense, and it was an even more ambitious method when computers were programmed by punch-cards and the protein databases were based on Dayhoff's three volumes of protein sequences [8].

### Blalock-Smith

This theory is called the *molecular recognition theory*; synonyms are *hydropathy complementarity *or *anti-complementarity theory*. It was based on the observation [22] that codons for hydrophilic and hydrophobic amino acids are generally complemented by codons for hydrophobic and hydrophilic amino acids, respectively. This is the case even when the complementary codons are read in the 3'→5"' direction. Peptides specified by complementary RNAs bind to each other with specificity and high affinity [23,24]. The theory turned out to be very fruitful in neuro-endocrine and immune research [25,26].

A very important observation is that antibodies against complementary antibodies also specifically interact with each other. Bost and Blalock [27] synthesized two complementary oligopeptides (i.e. peptides translated from complementary mRNAs, in opposing directions). The two peptides, Leu-Glu-Arg-Ile-Leu-Leu (LERILL), and its complementary peptide, Glu-Leu-Cys-Asp-Asp-Asp (ELCDDD), specifically recognized each other in radioimmunoassay. Antibodies were produced against both peptides. Each antibodies specifically recognized its own antigen. Using radioimmunoassays, anti-ELCDDD antibodies were shown to interact with ^125^I-labeled anti-LERILL antibodies but not with ^125^I-labeled control antibodies. More importantly, the interaction of the two antibodies could be blocked using either peptide antigen, but not by control peptides. Furthermore, ^125^I-labeled anti-LERILL binding to LERILL could be blocked with anti-ELCDDD antibody and vice versa. It was concluded therefore that antibody/antibody binding occurred at or near the antigen combining site, demonstrating that this was an idiotypic/anti-idiotypic interaction.

This experiment clearly showed the existence (and functioning) of an intricate network of complementary peptides and interactions. Much effort is being made to master this network and use it in protein purification, binding assays, medical diagnosis and therapy.

Recently, Blalock [28] has emphasized that nucleic acids encode amino acid sequences in a binary fashion with regard to hydropathy and that the exact pattern of polar and non-polar amino acids, rather than the precise identity of particular R groups, is an important driver for protein shape and interactions. Perfect codon complementarity behind the coding of interacting amino acids is no longer an absolute requirement for his theory.

**Figure 4:**
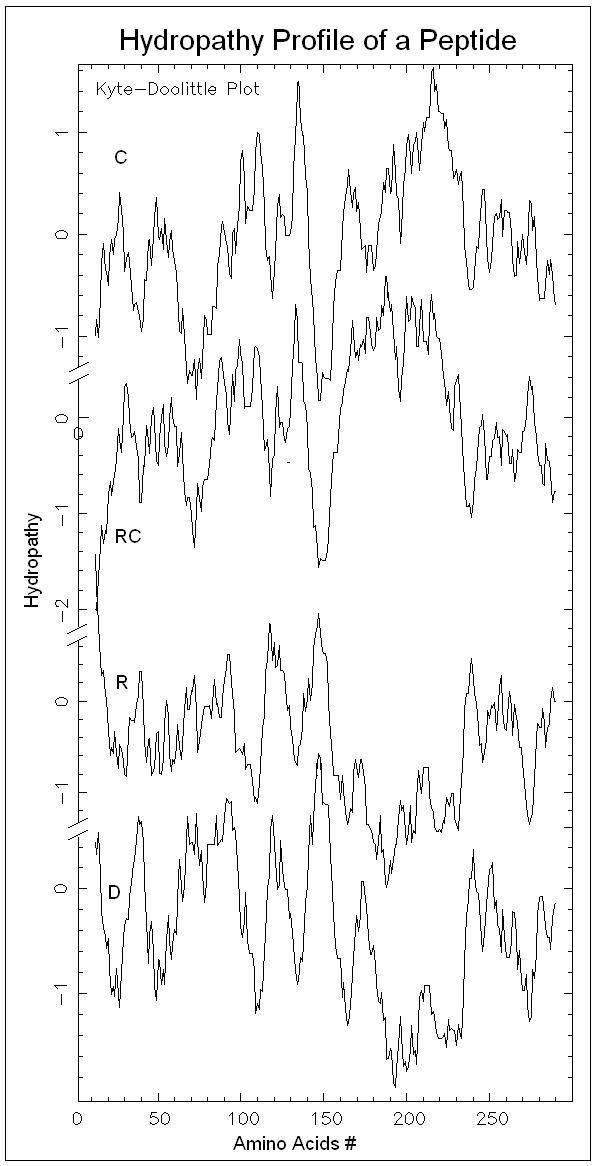
Hydropathy profile of a protein. An artificially constructed nucleic acid sequence was randomized and translated in the four possible directions (D, direct; RC, reverse-complementary; R, reverse; C, complementary). The D sequence was designed to contain equal numbers of the 20 amino acids.

Amino acids translated from complementary codons almost always show opposite hydropathy (Figure 4). However, the validity of hydrophobe-hydrophyl interactions remains unanswered.

### Root-Bernstein

Another amino acid pairing hypothesis was presented by Root-Bernstein [29,30]. He focused on whether it was possible to build amino acid pairs meeting standard criteria for bonding. He concluded that it was possible only in 26 cases (out of 210 pairs). Of these 26, 14 were found to be genetically encoded by perfectly complementary codons (read in the same orientation (5'→3'/3'→5') while in 12 cases mismatch was found at the wobble position of pairing codons.

### Siemion

There is a regular connection between activation energies (measured as enthalpies (Δ*H*^++^) and entropies (Δ*S*^++^) of activation for the reaction of 18 *N*"-hydroxysuccinimide esters of N-protected proteinaceous amino acids with *p*-anisidine) and the genetic code [31-33]. This periodic change of amino acid reactivity within the genetic code led him to suggest a peptide-anti-peptide pairing. This is rather similar to Root-Bernstein's hypothesis.

### Miller

Practical use is the best test of a theory. Technologies based on interacting proteins have a significant market in different branches of biochemistry, as well as in medical diagnostics and therapy. The Genetic Therapies Centre (GTC) at the Imperial College (London, UK) founded in 2001 with major financial support from a Japanese company, the Mitsubishi Chemical Corporation, and the UK charity, the Wolfson Foundation), is one of the first academic centers that are openly investing in Proteomic Code-based technologies. With the clear intention that their science "be used in the marketplace", Andrew Miller, the first director of GTC and co-founder of its first spin-off company, Proteom Ltd, is making major contributions to this field [34-38].

However, Miller and his colleagues came to realize that the amino acid pairs provided by perfectly complementary codons are not always the best pairs, and deviations from the original design sometimes significantly improved the quality of a protein-protein interaction. Therefore the current view of Miller is that there are "strategic pairs of amino acid residues that form part of a new, through-space two-dimensional amino acid interaction code (Proteomic Code). The proteomic code and derivatives thereof could represent a new molecular recognition code relating the 1D world of genes to the 3D world of protein structure and function, a code that could shortcut and obviate the need for extensive research into the proteome to give form and function to currently available genomic information (i.e., true functional genomics)".

### The Proteomic Code and the 3D structure of proteins

It is widely accepted that the 3D structures of proteins play a significant role in their specific interactions and function. The opposite is less obvious, namely that specific and individual amino acid pairs or sequences of these pairs might determine the foldings of proteins. Complementarity at the amino acid level in the proteins, and the corresponding internal complementarity within the coding mRNA (the Proteomic Code), raise the intriguing possibility that some protein folding information is present in the nucleic acids (in addition to or within the known and redundant genetic code). Real protein sequences show a higher frequency of complementarily coded amino acids than translations of randomized nucleotide sequences. [9-11]. The internal amino acid complementarity allows the polypeptides encoded by complementary codons to retain the secondary structure patterns of the translated strand (mRNA). Thus, genetic code redundancy could be related to evolutionary pressure towards retention of protein structural information in complementary codons and nucleic acid subsequences [39-44].

## Experimental evidence

Experiments based on the idea of a Proteomic Code usually start with a well-known receptor-ligand type protein interaction. A short sequence is selected (often <10 amino acids long) that is known or suspected to be involved in direct contact between the proteins in question (P-P/r). A complementary oligopeptide sequence is derived using the known mRNA sequence of the selected protein epitope, making a reverse complement of the sequence, translating it and synthesizing it.

The flow of the experiments is as follows:

(a) choose an interesting peptide;

(b) select a short, "promising" oligo-peptide epitope (P);

(c) find the true mRNA of P;

(d) reverse-complement this mRNA;

(e) translate the reverse-complemented mRNA into the complementary peptide (P/c);

(f) test P-P/c interaction (affinity, specificity);

(g) use P/c to find P-like sequences (for histochemistry, affinity purification);

(h) use P/c to generate antibodies (P/c_ab);

(i) test P/c_ab for its interaction with the P-receptor (P/r) and use it for (e.g.) labeling or affinity purification of P/r;

(j) use P_ab (as well as antibodies to P, P_ab) to find and characterize idiopathic (P_ab-P/c_ab) antibody reactions.

An encouraging feature of Proteomic-Code based technology is that the amino acid complementarity (information mirroring) does not stop with the P-P/c interaction but continues and involves even the antibodies generated against the original interacting domains; even P_ab-P/c_ab, i.e., antibodies against interacting proteins, will themselves contain interacting domains. They are idiotypes.

**Figure 5:**
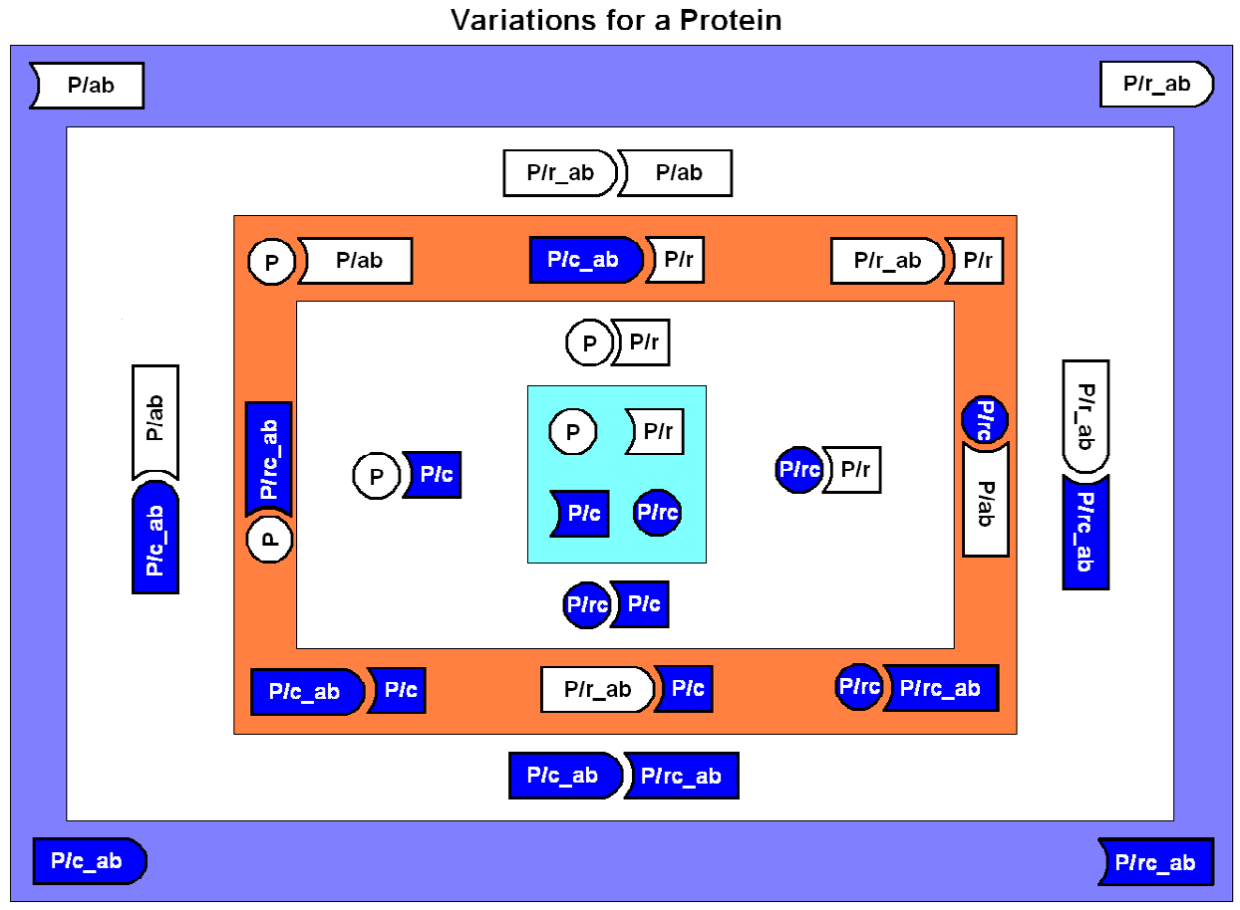
Variations for a protein. Experiments regarding the Proteomic Code are usually designed for the peptides and peptide interactions depicted in this figure. A peptide (P) naturally interacts with its receptor (P/r). Antibodies against this protein (P/ab) and its receptor (P/r_ab) might also be naturally present *in vivo *as part of the immune surveillance or might arise artificially. The Proteomic Code provides a method for designing artificial oligopeptides (P/c and P/rc) that can interact strongly with the receptor and its ligand. P and P/c as well as Pr and P/rc are expressed from complementary nucleic acid sequences. It is possible to raise antibodies against P/c (P/c_ab) and P/rc (P/rc_ab).

Peptides and interactions involved in Proteomic Code-based experiments are summarized in Figure 5.

An impressive example of this technology and its potential is given by Bost and Blalock [27] (described above), It is reviewed by Heal et al. [37] and McGuian [45]. A collection of examples [see Additional file 1] presents a number of experiments of this kind.

Some experiments or types of experiments require further attention.

The *antisense homology box*, a new motif within proteins that encodes biologically active peptides, was defined by Baranyi and coworkers around 1995. They used a bioinformatics method for a genome-wide search of peptides encoded by complementary exon sequences. They found that amphiphilic peptides, approximately 15 amino acids in length, and their corresponding antisense peptides exist within protein molecules. These regions (termed antisense homology boxes) are separated by approximately 50 amino acids. They concluded that because many sense-antisense peptide pairs have been reported to recognize and bind to each other, antisense homology boxes may be involved in folding, chaperoning and oligomer formation of proteins. The frequency of peptides in antisense homology boxes was 4.2 times higher than expected from random sequences (*p *< 0.001) [46].

They successfully confirmed their suggestion by experiments. The antisense homology box-derived peptide CALSVDRYRAVASW, a fragment of the human endothelin A receptor, proved to be a specific inhibitor of endothelin peptide (ET-1) in a smooth muscle relaxation assay. The peptide was also able to block endotoxin-induced shock in rats. The finding of an endothelin receptor inhibitor among antisense homology box-derived peptides indicates that searching proteins for this new motif may be useful in finding biologically active peptides [47-49].

A bioinformatics experiment similar to Baranyi's was performed by Segerstéen et al. [50]. They tested the hypothesis that nucleic acids, encoding specifically-interacting receptor and ligand proteins contain complementary sequences. Human insulin mRNA (HSINSU) contained 16 sequences that were 23.8 ± 1.4 nucleotides long and were complementary to the insulin receptor mRNA (HSIRPR, 74.8 ± 1.9% complementary matches, *p *< 0.001 compared to randomly-occurring matches). However, when 10 different nucleic acids (coding proteins not interacting with the insulin receptor) were examined, 81 additional sequences were found that were also complementary to HSIRPR. Although the finding of short complementary sequences was statistically highly significant, we concluded that this is not specific for nucleic acid coding of specifically interacting proteins.

There are two kinds of antisense technologies based on the complementarity of nucleic acids: (a) when the production of a protein is inhibited by an oligonucleotide sequence complementary to its mRNA; this is a pre-translational modification and it usually requires transfer of nucleic acids into the cells; (b) when the biological effect of an already complete protein is inhibited by another protein translated from its complementary mRNA; this is a post-translational modification and does not block the synthesis of a protein.

Many experiments [see Additional file 1] indicate that antisense proteins inhibit the biological effects of a protein. This suggests the possibility of *antisense protein therapy*. The P-P/c reaction is in many respects similar to the antigen-antibody reaction, therefore the potential of antisense protein therapy is expected to be similar to the potential of antibody therapy (passive immunization against proteinaceous toxins, such as bacterial toxins, venoms, etc.). However, antisense peptides are much smaller than antibodies (MW as little as ~1000 Da compared to IgG ~155 kDa). This means that antisense proteins are easy to manufacture in vitro; antibodies are produced in living animals (with non-human species characteristics). However, the small size is expected to have the disadvantage of a lower *K*_d _and a shorter biological half-life.

Immunization with complementary peptides produces antibodies (P/c_ab) as with any other protein. These antibodies contain a domain that is similar to the original protein (P) and specifically binds to the receptor of the original protein (P/r). This property is effectively used for affinity purification or immuno-staining of receptors. The P/c_ab is able to mimic or antagonize the in vivo effect of P by binding to its receptor. This property has the desired potential to treat protein-related diseases such as many pituitary gland-related diseases. A vision might be to treat, for example, pituitary dwarfism, with immunization against growth hormone complementary peptide (GH/c), or Type I diabetes with immunization against insulin/c peptide.

### Reverse but not complementary sequences

The biochemical process of transcription and translation is unidirectional, 5'→3', and reversion does not exist. However, there are many examples of sequences present in the genome (in addition to direct reading) in reverse orientation, and if expressed (in the usual 5'→3' direction) they produce mRNA and proteins that are, in effect, reversely transcribed and reversely translated.

An interesting observation is that direct and reverse proteins often have very similar binding properties and related biological effects even if their sequence homology is very low (<20%). For example, growth hormone-releasing hormone (GHRH) and the reverse GNRH specifically bind to the GHRH receptor on rat pituitary cells and to polyclonal anti-GHRH antibody in ELISA and RIA procedures although they share only 17% sequence similarity and they are antagonists in in vitro stimulation of GH RNA synthesis and in vitro and in vivo GH release from pituitary cells [51].

The same phenomenon is observed in complementary sequences. A peptide expressed by complementary mRNA often specifically interacts with proteins expressed by the direct mRNA and it does not matter if they are read in the same or opposite directions. A possible explanation is that many codons are actually symmetrical and have the same meaning in both directions of reading. The physico-chemical properties of amino acids are preferentially determined by the 2nd (central) codon letter [52] so the physico-chemical pattern of direct and reverse sequences remains the same. In addition, I found that protein structural information is also carried by the 2nd codon letters [53].

### Controversies regarding the original Proteomic Codes

All proteomic codes before 2006 required perfect complementarity, even if it was noticed that the "biophysical and biological properties of complementary peptides can be improved in a rational and logical manner where appropriate" [36].

- Expression of the antisense DNA strand was simply not accepted before large scale genome sequences confirmed that genes are about equally distributed on both strands of DNA in all organisms containing dsDNA.

- Spatial complementarity is difficult to imagine between longer amino acid sequences, because the natural, internal folding of proteins will prohibit it in most cases.

- Usually, residues with the same polarity are attracted to each other, because hydrophobes prefer a hydrophobic environment and lipophobes prefer lipophobic neighbors. Amphipathic interactions seem artificial to most chemists.

- Only complementary (but not reversed) sequences were found as effective as direct ones. This requires 3'→5' translation, which is normally prohibited.

- The results are inconsistent; it works for some proteins but not for others; it is necessary to improve results, e.g., "M-I pair mutagenesis" [36].

- Protein 3D structure and interactions are thought to be arranged on a larger scale than individual amino acids.

- The number of possible amino acid pairs is 20 × 20/2 = 200. The number of perfect codons is 64, i.e., about a third of the number expected. This means that two-thirds of amino acid pairs are impossible to encode in perfectly complementary codons.

• are these amino acid pairs not derived from complementary codons at all?

• are these amino acid pairs derived from imperfectly complementary codons?

## Development of the second generation Proteomic Code

What did we learn about the Proteomic Code during its first 25 years (1981–2006)? My first and most important lesson is that I realize how terribly wrong it was (and is) to believe in scientific dogmas, such as sense vs nonsense DNA strands. It is almost unbelievable today that many of us were able to see a difference between two perfectly symmetrical and structurally identical strands.

We were able to provide multiple independent strands of convincing evidence that the concept of the Proteomic Code is valid. At the same time we had to understand that the first concepts – based on perfect complementarity of codons behind interacting amino acids – were imperfect. There is protein folding information in the nucleic acids – in addition to or within the redundant genetic code – but it is unclear how is it expressed and interpreted to form the 3D protein structure.

A major physico-chemical property, the hydropathy of amino acids, is encoded by the codons. Proteins translated from direct and reverse as well as from complementary and reverse-complementary strands have the same hydropathic profiles. This is possible only if the amino acid hydropathy is related to the second, central codon letter.

There is a clear indication that some biological information exists in multiple complementary (mirror) copies: DNA-DNA/c→RNA-RNA/c→protein-protein/c→IgG-IgG/c.

Some theoretical considerations and research that led to the suggestion of the 2nd generation Proteomic Codes are now reviewed.

### Construction of a Common Periodic Table of Codons and Amino Acids

The Proteomic Code revitalizes a very old dilemma and dispute about the origin of the genetic code, represented by Carl Woese and Francis Crick. Is there any logical connection between any properties of an amino acid on the one hand and any properties of its genetic code on the other?

Carl Woese [54] argued that there was stereochemical matching, i.e., affinity, between amino acids and certain triplet sequences. He therefore proposed that the genetic code developed in a way that was very closely connected to the development of the amino acid repertoire, and that this close biochemical connection is fundamental to specific protein-nucleic acid interactions.

Crick [55] considered that the basis of the code might be a "frozen accident", with no underlying chemical rationale. He argued that the canonical genetic code evolved from a simpler primordial form that encoded fewer amino acids. The most influential form of this idea, "code co-evolution," proposed that the genetic code co-evolved with the invention of biosynthetic pathways for new amino acids [56].

**Figure 6:**
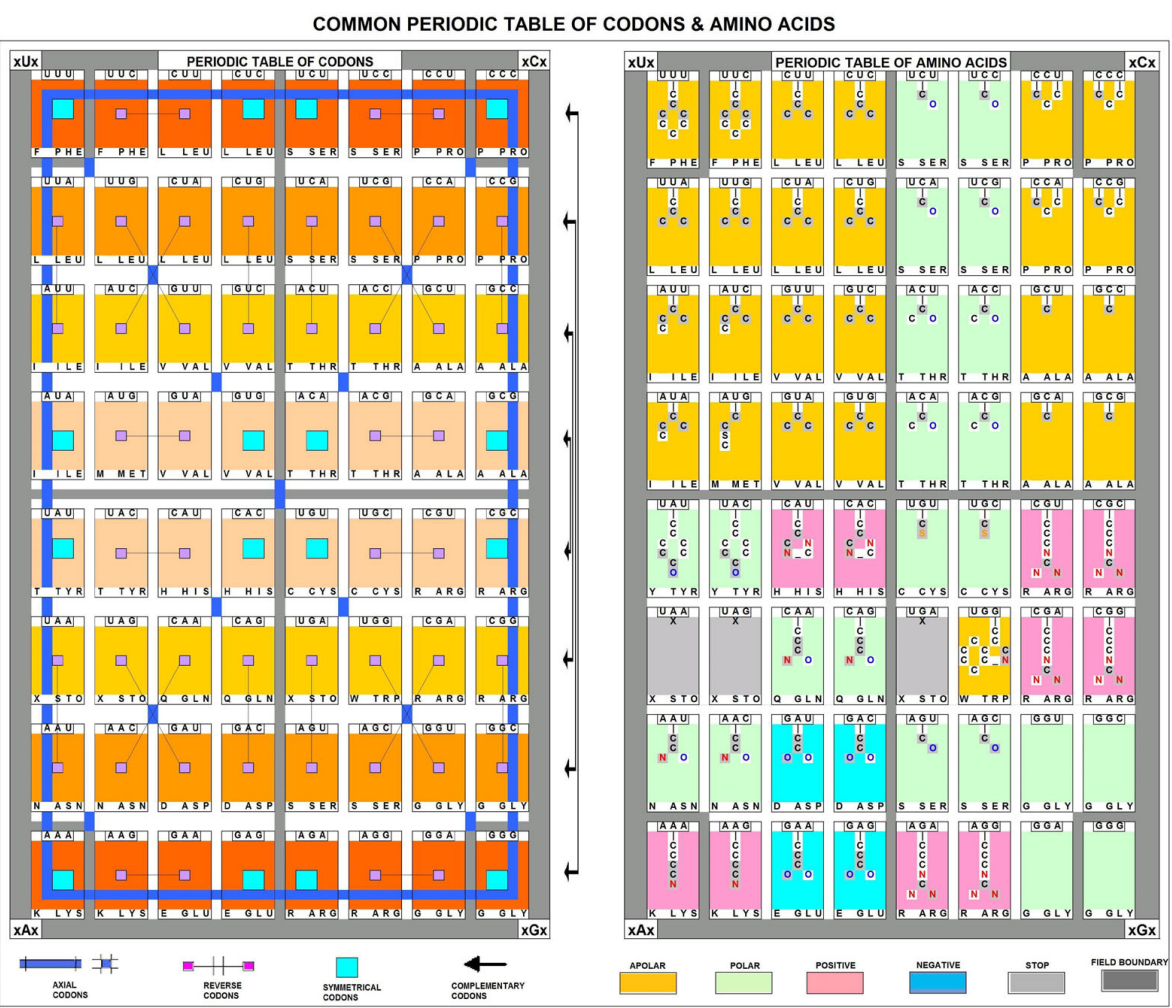
Common Periodic Table of Codons & Amino Acids (modified from [52]).

A periodic table of codons has been designed in which the codons are in regular locations. The table has four fields (16 places in each), one with each of the four nucleotides (A, U, G, C) in the central codon position. Thus, AAA (lysine), UUU (phenylalanine), GGG (glycine) and CCC (proline) are positioned in the corners of the fields as the main codons (and amino acids). They are connected to each other by six axes. The resulting nucleic acid periodic table shows perfect axial symmetry for codons. The corresponding amino acid table also displaces periodicity regarding the biochemical properties (charge and hydropathy) of the 20 amino acids, and the positions of the stop signals. Figure 6 emphasizes the importance of the central nucleotide in the codons, and predicts that purines control the charge while pyrimidines determine the polarity of the amino acids.

**Figure 7:**
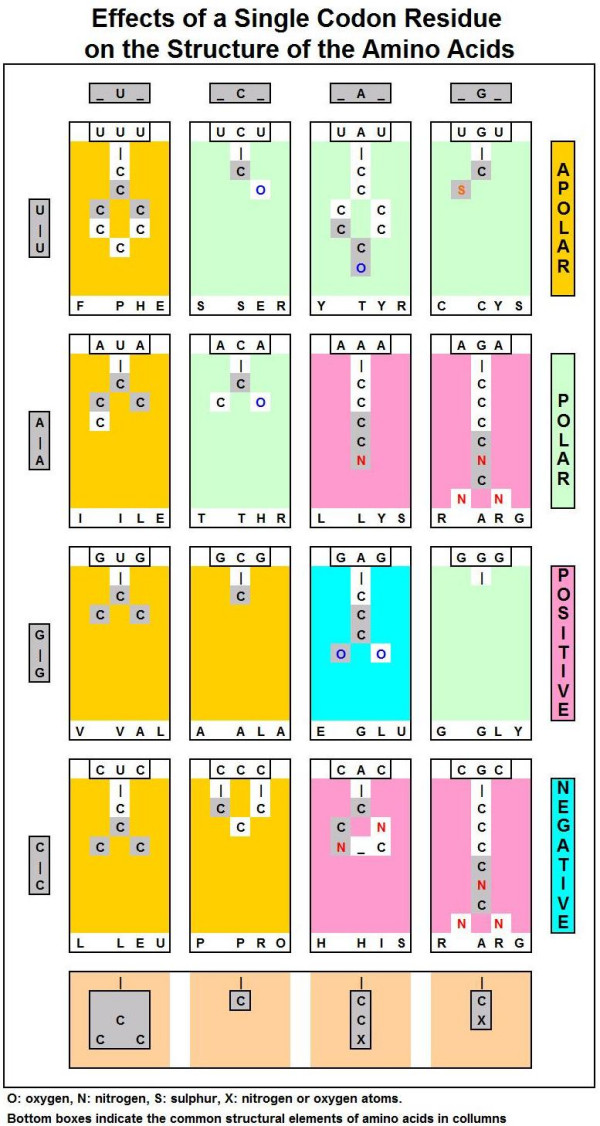
Effects of a single codon residue on the structure of the amino acids.

In addition to this correlation between the codon sequence and the physico-chemical properties of the amino acids, there is a correlation between the central residue and the chemical structure of the amino acids. A central uridine correlates with the functional group -C(C)_2_-; a central cytosine correlates with a single carbon atom, in the C_1 _position; a central adenine coincides with the functional groups -CC = N and -CC = O; and finally a central guanine coincides with the functional groups -CS, -C = O, and C = N, and with the absence of a side chain (glycine). (Figure 7)

I interpret these results as a clear-cut answer for the Woese vs Crick dilemma: there is a connection between the codon structure and the properties of the coded amino acids. The second (central) codon base is the most important determinant of the amino acid property. It explains why the reading orientation of translation has so little effect on the hydropathy profile of the translated peptides. Note that 24 of 32 codons (U or C in the central position) code apolar (hydrophobic) amino acids, while only 1 of 32 codons (A or G in the central position) codes non-apolar (non-hydrophobic, charged or hydrophilic) amino acids. It explains why complementary amino acid sequences have opposite hydropathy, even if the binary hydropathy profile is the same.

### The physico-chemical compatibility of amino acids in the Proteomic Code

Complementary coding of two amino acids is not a guarantee per se of the special co-location (or interaction) of these amino acids within the same or between two different peptides. Some kind of physico-chemical attraction is also necessary. The most fundamental properties to consider are, of course, the size, charge and hydropathy. Mekler and I suggested size compatibility [9-11,20], obviously under the influence of the known size complementarity of the Watson-Crick base pairs. Blalock emphasized the importance of hydropathy, or rather amphipathy (which makes some scientists immediately antipathic). Hydrophobic residues like other hydrophobic residues and hydrophilic residues like hydrophilic residues. Hydrophyl and hydrophobe residues have difficulties to share the same molecular environment.

**Figure 8:**
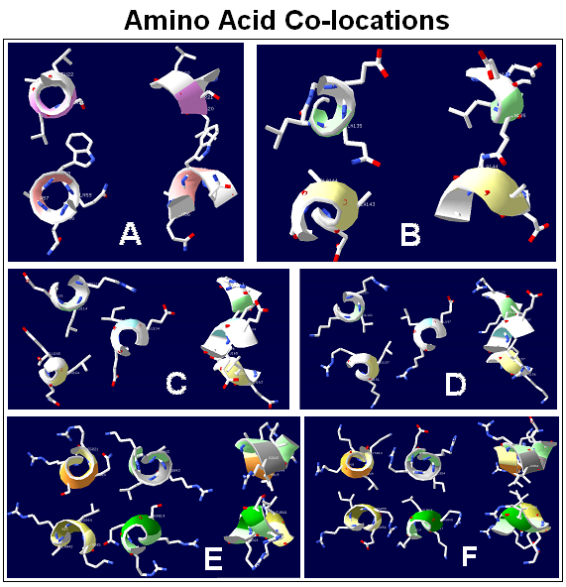
Amino acid co-locations. Randomly selected amino acid contacts from real proteins. The interactions between amino acid residues from 2 (A, B) 3 (C, D) and 4 (E, F) parallel alpha helices are perpendicular to the peptide backbones (helices). The orientations of residues show considerable variation; some are located side-by-side, others are end-to-end.

Visual studies of the 3D structures of proteins give some ideas of how interacting interfaces look (Figure 8):

- the interacting (co-locating) sequences are short (1–10 amino acid long);

- the interacting (co-locating) sequences are not continuous; there are many mismatches;

- the orientations of co-locating residues are often not the same (not parallel);

- the contact between co-locating residues might be side-to-side or top-to-top.

This is clearly a different picture from the base-pair interactions in a dsDNA spiral. Alpha-helices and beta-sheets are regular structures, which make their amino acid residues periodically ordered. Many residues are parallel to each other and W-C-like interactions are not impossible. But is it really the explanation for specific residue interactions?

### SeqX

The interacting residues of protein and nucleic acid sequences are close to each other; they are co-located. Structure databases (e.g., Protein Data Bank, PDB and Nucleic Acid Data Bank, NDB) contain all the information about these co-locations; however, it is not an easy task to penetrate this complex information. We developed a JAVA tool, called SeqX, for this purpose [57]. The SeqX tool is useful for detecting, analyzing and visualizing residue co-locations in protein and nucleic acid structures. The user:

(a) selects a structure from PDB;

(b) chooses an atom that is commonly present in every residue of the nucleic acid and/or protein structure(s);

(c) defines a distance from these atoms (3–15 Å).

**Figure 9:**
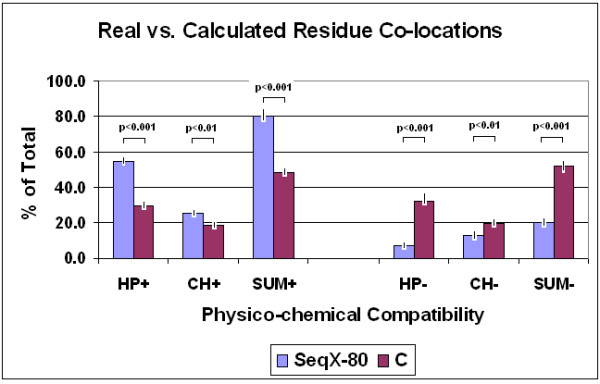
Real vs calculated residue co-locations (from [57]). The relative frequency of real residue co-locations was determined by SeqX in 80 different protein structures and compared to the relative frequency of calculated co-locations in artificial, random protein sequences (C). The 200 possible residue pairs provided by the 20 amino acids were grouped into 4 subgroups on the basis of their mutual physico-chemical compatibility, i.e., favored (+) and un-favored (-) in respect of hydrophobicity and charge. (HP+, hydrophobe-hydrophobe and lipophobe-lipophobe; HP-, hydrophobe-lipophobe; CH+, positive-negative and hydrophobe-charged; CH-: positive-positive, negative-negative and lipophobe-charged interactions). The bars represent the mean ± SEM (*n *= 80 for real structures and *n *= 10 for artificial sequences). Student's *t*-test was applied to evaluate the results.

The SeqX tool then detects every residue that is located within the defined distances from the defined "backbone" atom(s); provides a dot-plot-like visualization (residues contact map); and calculates the frequency of every possible residue pair (residue contact table) in the observed structure. It is possible to exclude ± 1–10 neighbor residues in the same polymeric chain from detection, which greatly improves the specificity of detections (up to 60% when tested on dsDNA). Results obtained on protein structures show highly significant correlations with results obtained from the literature (*p *< 0.0001, *n *= 210, four different subsets). The co-location frequency of physico-chemically compatible amino acids is significantly higher than is calculated and expected for random protein sequences (*p *< 0.0001, *n *= 80) (Figure 9).

These results gave a preliminary confirmation of our expectation that physico-chemical compatibility exists between co-locating amino acid pairs. Our findings do not support any significant dominance of amphipathic residue interactions in the structures examined.

### Amino acid size, charge, hydropathy indices and matrices for protein structure analysis

It was necessary to look more closely at the physico-chemical compatibility of co-locating amino acids [58].

**Figure 10:**
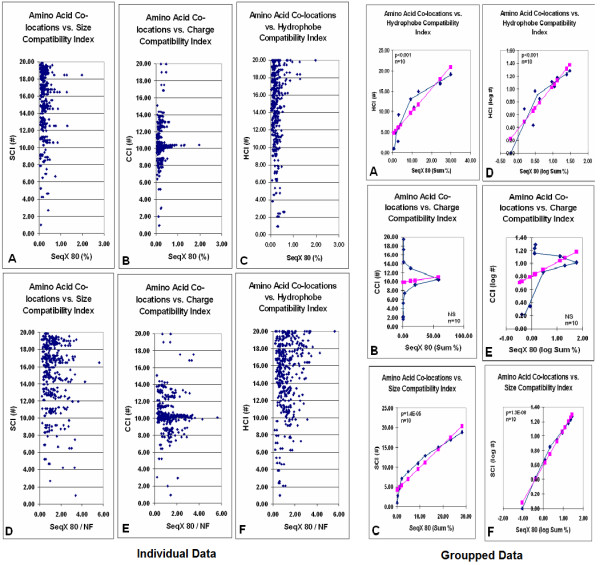
Amino acid co-locations vs size, charge, and hydrophobe compatibility indexes (modified from [58]). Individual data (left) Average propensity of the 400 different amino acid co-locations in 80 different protein structures (SeqX 80) are plotted against size, charge and hydrophobe compatibility indexes (SCI, CCI, HCI). The original "row" values are indicated in (A-C). The SeqX 80 values were corrected by the co-location values, which are expected only by chance in proteins where the amino acid frequency follows the natural codon frequency (NF) (D-F). Individual data (left) were divided into subgroups and summed (Sum) (Groupped data, right). The group averages are connected by the blue lines while the pink symbols and lines indicate the calculated linear regression.

We indexed the 200 possible amino acid pairs for their compatibility regarding the three major physico-chemical properties – size, charge and hydrophobicity – and constructed size, charge and hydropathy compatibility indices (SCI, CCI, HCI) and matrices (SCM, CCM, HCM). Each index characterized the expected strength of interaction (compatibility) of two amino acids by numbers from 1 (not compatible) to 20 (highly compatible). We found statistically significant positive correlations between these indices and the propensity for amino acid co-locations in real protein structures (a sample containing a total of 34,630 co-locations in 80 different protein structures): for HCI, *p *< 0.01, *n *= 400 in 10 subgroups; for SCI, *p *< 1.3E-08, *n *= 400 in 10 subgroups; for CCI, *p *< 0.01, *n *= 175). Size compatibility between residues (well known to exist in nucleic acids) is a novel observation for proteins (Figure 10).

**Figure 11:**
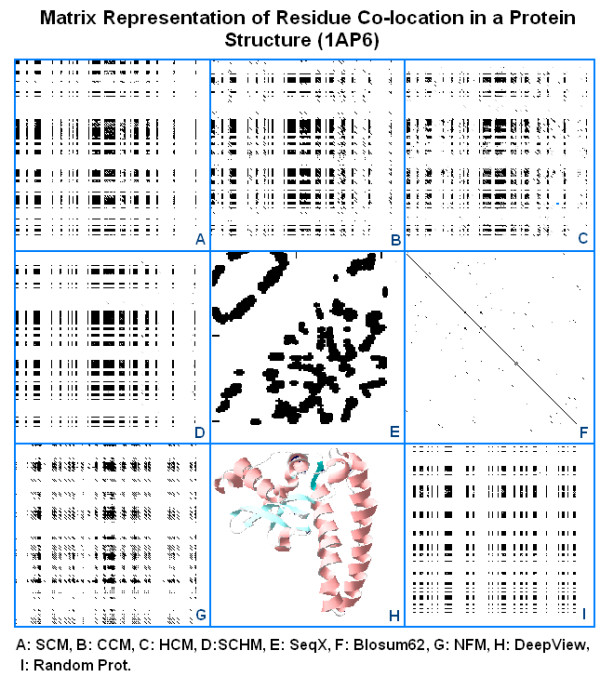
Matrix representation of residue co-locations in a protein structure (1AP6) (modified from [58]). A protein sequence (1AP6) was compared to itself with DOTLET using different matrices, SCM (A), CCM (B), HCM (C), the combined SCHM (D) and NFM (G) and Blosum62 (F). Comparison of randomized 1AP6 using SCHM is seen in (I). The 2D (SeqX Residue Contact Map) and 3D (DeepView/Swiss-PDB Viewer) views of the structure are illustrated in (E) and (H). The black/gray parts of the dot-plot matrices indicate the respective compatible residues, except the Blosum62 comparison (F), where the diagonal line indicates the usual sequence similarity. The dot-plot parameters are otherwise the same for all matrices.

We tried to predict or reconstruct simple 2D representations of 3D structures from the sequence using these matrices by applying a dot-plot-like method. The locations and patterns of the most compatible subsequences were very similar or identical when the three fundamentally different matrices were used, which indicates the consistency of physico-chemical compatibility. However, it was not sufficient to choose one preferred configuration between the many possible predicted options (Figure 11).

Indexing of amino acids for major physico-chemical properties is a powerful approach to understanding and assisting protein design. However, it is probably insufficient itself for complete ab initio structure prediction.

### Anfinsen's thermodynamic principle and the Proteomic Code

The existence of physico-chemical compatibility of co-locating amino acids even on the single residue level is, of course, a necessary support for the Proteomic Code. At the same time, it raises the possibility that protein structure might be predicted from the primary amino acid sequence (de novo, ab initio prediction) and the location of physico-chemically compatible amino acid residues in the sequence. This idea is in line with a dominating statement about protein folding: Anfinsen's thermodynamic principle states that all information necessary to form a 3D protein structure is present in the protein sequence [59].

Attempts were made to use the three different matrices in a dot plot to predict the place and extent of the most likely residue co-locations. This visual, non-quantitative method indicated that the three very different matrices located very similar residues and subsequences as potential co-location places. No single diagonal line was seen in the dot-plot matrices, which is the expected signature of sequence similarity (or compatibility in our case). Instead, block-like areas indicated the place and extent of predicted sequence compatibilities. It was not possible to reconstruct a real map of any protein 2D structure (Figure 11) [60].

This experience with the indices provides arguments for as well as against Anfinsen's theorem. The clear-cut action of basic physico-chemical laws at the residue level is well in line with the lowest free energy requirement of the law of entropy. Furthermore, this obvious presence of physico-chemical compatibility is easy to understand, even from an evolutionary perspective. In evolution, sequence changes more rapidly than structure; however, many sequence changes are compensatory and preserve local physico-chemical characteristics. For example, if, in a given sequence, an amino acid side chain is particularly bulky with respect to the average at a given position, this might have been compensated in evolution by a particularly small side chain in a neighboring position, to preserve the general structural motif. Similar constraints might hold for other physico-chemical quantities such as amino acid charge or hydrogen bonding capacity [61].

We were not able to reconstruct any structure using our indices. There are massive arguments against Anfinsen's principle:

(1) The connection between primary, secondary and tertiary structure is not strong, i.e., in evolution, sequence changes more rapidly than structure. Structure is often conserved in proteins with similar function even when sequence similarity is already lost (low structure specificity to define a sequence). Identical or similar sequences often result in different structures (low sequence specificity to define a structure).

(2) An unfolded protein has a vast number of accessible conformations, particularly in its residue side chains. Entropy is related to the number of accessible conformations. This problem is known as the Levinthal paradox [62].

(3) The energy profile characteristics of native and designed proteins are different. Native proteins usually show a unique and less stable profile, while designed proteins show lower structural specificity (many different possible structures) but high stability [63].

(4) The entropy minimum is a statistical minimum. The conformation entropy change of the whole molecule is the sum of local (residue level) conformation entropy changes and it permits many different local conformation variations to co-exist. It is doubtful whether structural variability (heterogeneity, instability) is compatible with the function (homogeneity, stability) of a biologically active molecule.

The present experiments do not decide the "fate" of Anfinsen's dogma; however, they show that the number of possible co-locating places is too large, and searching this space poses a daunting optimization problem. It is not realistic to expect the ab initio prediction of only one single structure from one primary protein sequence. The development of a prediction tool for protein structure (like an mfold for nucleic acids [64], that provides only a few hundred most likely (thermodynamically most optimal) structure suggestions per protein sequence seems to be closer. It is likely that SCM, CCI and HCM (or similar matrices) will be essential elements of these tools.

Additional folding information might be necessary (in addition to that carried in the protein primary sequence) to be able to create a unique protein structure. Such information is suspected to be present in the redundant genetic code [65-67].

### Protein structure and the functional asymmetry of the codons

I agree with Levinthal that the Anfinsen's thermodynamic principle is insufficient.

There are two potential, external sources of additional and specific protein folding information: (a) the chaperons (other proteins that assist in the folding of proteins and nucleic acids [70]); and (b) the protein-encoding nucleic acid sequences themselves (which are the templates for protein syntheses but are not defined as chaperons).

The idea that the nucleotide sequence itself could modulate translation and hence affect the co-translational folding and assembly of proteins has been investigated in a number of studies [71,72]. Studies on the relationships between synonymous codon usage and protein secondary structural units are especially popular [67,73,74]. The genetic code is redundant (61 codons encode 20 amino acids) and as many as 6 synonymous codons can encode the same amino acid (Arg, Leu, Ser). The "wobble" base has no effect on the meaning of most codons, but codon usage (wobble usage) is still not randomly defined [75,76] and there are well known, stable species-specific differences in codon usage. It seems logical to search for some meaning (biological purpose) of the wobble bases and try to associate them with protein folding.

Another observation concerning the code redundancy dilemma is that there is a widespread selection (preference) for local RNA secondary structure in protein coding regions [77]. A given protein can be encoded by a large number of distinct mRNA species, potentially allowing mRNAs to optimize desirable RNA structural features simultaneously with their protein coding function. The immediate question is whether there is some logical connection between the possible, optimal RNA structures and the possible, optimal biologically active protein structures.

Single-stranded RNA molecules can form local secondary structures through the interactions of complementary segments. W-C base pair formation lowers the average free energy, d*G*, of the RNA and the magnitude of change is proportional to the number of base pair formations. Therefore the free folding energy (FFE) is used to characterize the local complementarity of nucleic acids [77]. The free folding energy is defined as FFE = {(d*G*_shuffled _- d*G*_native_)/*L*} × 100, where *L *is the length of the nucleic acid, i.e., the free energy difference between native and shuffled (randomized) nucleic acids per 100 nucleotides. Higher positive values indicate stronger bias towards secondary structure in the native mRNA, and negative values indicate bias against secondary structure in the native mRNA.

We used a nucleic acid secondary structure predicting tool, mfold [64], to obtain d*G *values and the lowest d*G *was used to calculate the FFE. mfold also provided the folding energy dot-plots, which are very useful for visualizing the energetically most favored structures in a 2D matrix.

A series of JAVA tools were used: SeqX to visualize the protein structures in 2D as amino acid residue contact maps [57]; SeqForm for selection of sequence residues in predefined phases (every third in our case) [78]; SeqPlot for further visualization and statistical analyses of the dot-plot views [79]; Dotlet as a standard dot-plot viewer [80]. Structural data were downloaded from PDB [81], NDB [82], and from a wobble base oriented database called Integrated Sequence-Structure Database (ISSD) [83].

Structures were generally randomly selected in regard to species and biological function (a few exceptions are mentioned below). Care was taken to avoid very similar structures in the selections. A propensity for alpha helices was monitored during selection and structures with very high and very low alpha helix content were also selected to ensure a wide range of structural representation.

Linear regression analyses and Student's *t*-tests were used for statistical analyses of the results.

**Figure 12:**
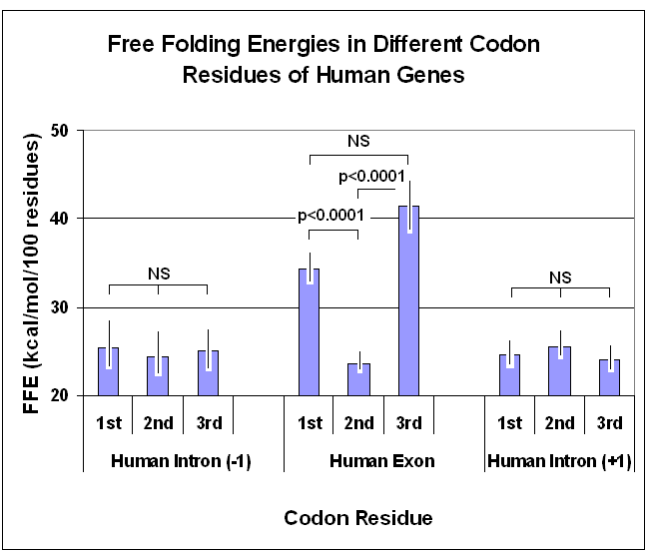
Free folding energies (FFE) in different codon residues of human genes. The coding sequences (exons) of 18 human hormone genes and the preceding (-1) and following (+1) sequences (introns) were phase separated into three subsequences each corresponding to the 1st, 2nd and 3rd codon positions in the coding sequence. The d*G *values were determined by mfold and the FFE was calculated. Each bar represents the mean ± SEM, *n *= 18.

Observations were made on human peptide hormone structures. This group of proteins is very well defined and annotated, the intron-exon boundaries are known and even intron data are easily accessible. The coding sequences were phase separated by SeqForm into three subsequences, each containing only the 1st, 2nd or 3rd letters of the codons. Similar phase separation was made for intronic sequences immediately before and after the exon. There are, of course, no known codons in the intronic sequences, therefore we continued the same phase that we applied for the exon, assuming that this kind of selection is correct, and maintained the name of the phase denotation even for non-coding regions. Subsequences corresponding to the 1st and 3rd codon letters in the coding regions had significantly higher FFEs than subsequences corresponding to the 2nd codon letters. No such difference was seen in non-coding regions (Figure 12).

**Figure 13:**
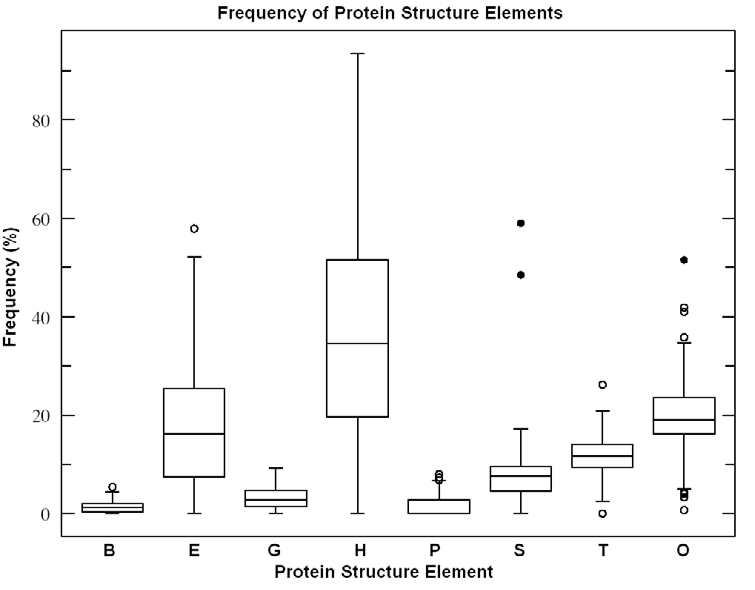
Frequency of protein structure elements. Box plot representation of protein secondary structure elements in 81 structures. *L *= 317 ± 20 (mean ± SEM, *n *= 81). Secondary structure codes: H, alpha helix; B, residue in isolated beta bridge; E, extended strand, participates in beta ladder; G, 3-helix (3/10 helix); I, 5 helix (pi helix); P, polyproline type II helix (left-handed); T, hydrogen bonded turn; S, bend.

In a larger selection of 81 different protein structures, the corresponding protein and coding sequences were used to extend the observations. These 81 proteins represented different (randomly selected) species and different (also randomly selected) protein functions and therefore the results might be regarded as more generally valid. The propensity for different secondary structure elements was recorded (as annotated in different databases) (Figure 13).

**Figure 14:**
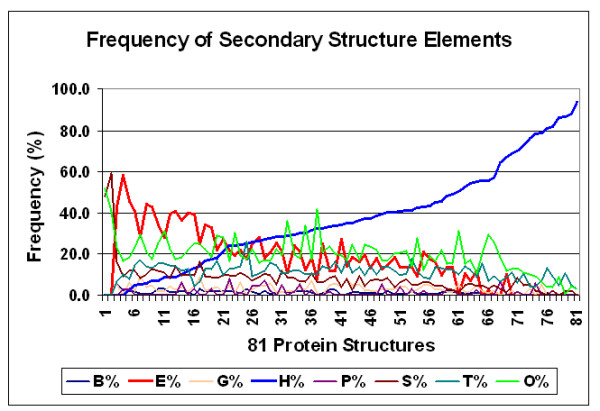
Frequency of secondary structure elements. The propensity of different structural elements in 81 different proteins is shown. *L *= 317 ± 20 (mean ± SEM, *n *= 81). Secondary structure codes: H, alpha helix; B, residue in isolated beta bridge; E, extended strand, participates in beta ladder; G, 3-helix (3/10 helix); I, 5 helix (pi helix); P, polyproline type II helix (left-handed); T, hydrogen bonded turn; S, bend.

**Figure 15:**
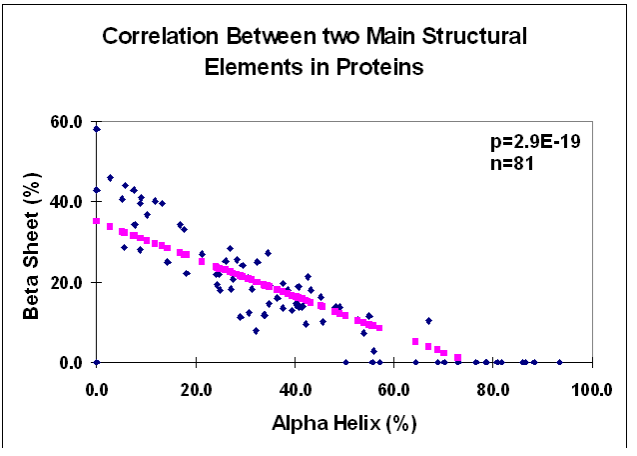
Correlation between two main structural elements in proteins. Data were taken from Figure 14 (H, alpha helix; E, beta sheet).

The proportion of alpha helices varied from 0 to 90% in the 81 proteins and showed a significant negative correlation to the proportion of beta sheets (Figures 14 and 15).

**Figure 16:**
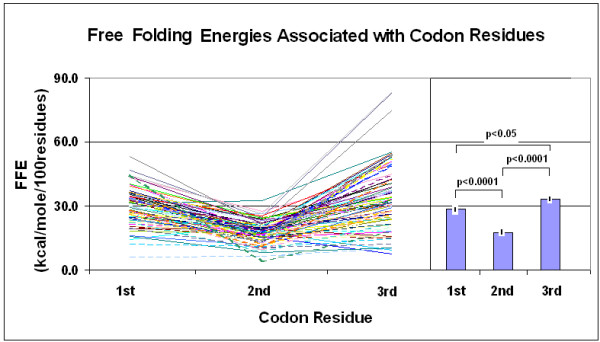
Free folding energies associated with codon residues (Free folding energies (FFE) were determined in phase-selected subsequences of 81 different protein protein-encoding nucleic acids. The lines indicate individual values (left part of the figure), while the bars (right part of the figure) indicate the mean ± SEM (*n *= 81).

The original observation made on human protein hormones, that significantly more free folding energy is associated with the 1st and 3rd codon residues than with the 2^nd^, was confirmed on a larger and more heterogeneous protein selection. A significant difference was apparent even between the 1st and 3rd residues in this larger selection (Figure 16).

**Figure 17:**
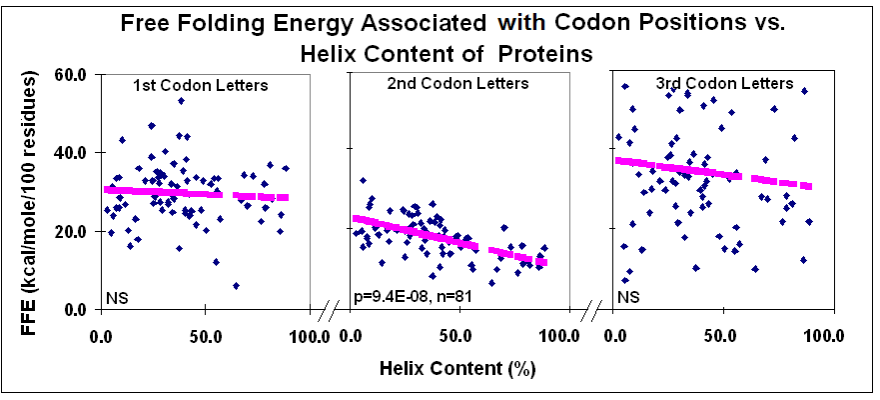
Free folding energy associated with codon positions vs helix content of proteins. Linear regression analyses; pink symbols represent the linear regression line.

**Figure 18:**
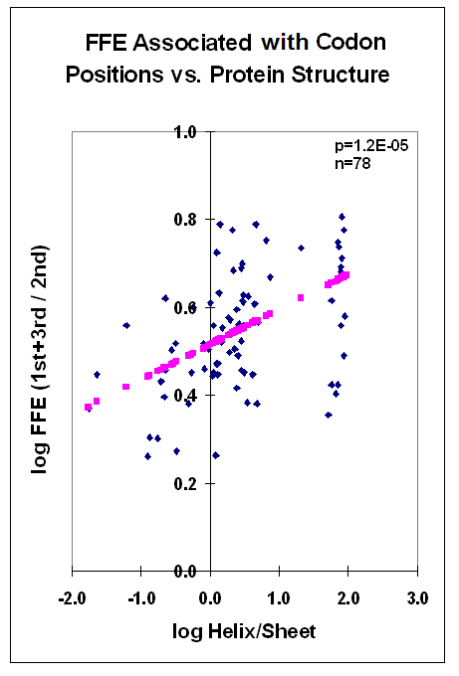
FFE associated with codon positions vs protein structure. Same data as in Figure 17 after calculating ratios and log transformation. Linear regression analyses; pink symbols represent the linear regression line.

**Figure 19:**
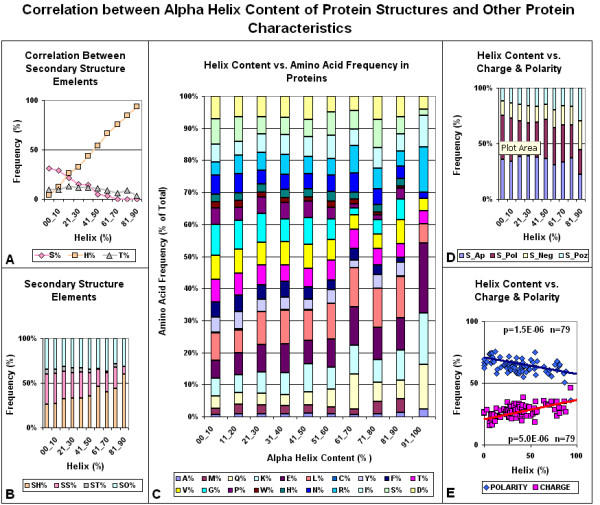
Correlation between alpha helix content of protein structure and other protein characteristics. The alpha helix content of 80 protein structures was compared to the frequency of other major structural elements (A,B), the frequency of individual amino acids (C) and the frequency of charged and hydrophobic residues (D,E). (A) The correlation between helix (H), beta sheet (S) and turn (T); (B) the proportions between the sum of helices (SH), beta strands (SS), turns (ST) and all other structural elements (TO). (D) The proportion between the sums of apolar (S_Ap), polar (S_Pol), negatively charged (S_Neg) and positively charged (S_Poz) amino acids. (E) The linear regression analysis correlations between helix content and the percentages of polar+apolar (Polarity) and positively+negatively charged (Charge) residues.

**Figure 20:**
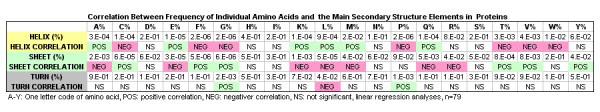
Correlation between frequency of individual amino acids and the main secondary structure elements in proteins. See text for explanation.

There is a correlation between the protein structure and the FFE associated with codon residues. The correlation is negative between the FFEs associated with the 2^nd ^(middle) codon residues and the alpha helix content of the protein structure. The correlation is especially significant when the FFE ratios are compared to the helix/sheet ratios (Figures 17 and 18). The alpha helix is the most abundant structural element in proteins. It shows negative correlation to the frequency of the second most prominent protein structure, the beta sheet. The propensity for some amino acids and the major physico-chemical characteristics (charge and polarity) show significant correlation (positive or negative) to this structural feature. We include statistical analyses of alpha helix content and other protein characteristics to show the complexity behind the term "alpha helix" and to demonstrate the insecurity in interpreting any correlation to this structural feature (Figures 19 and 20). Detailed analyses of these data are outwith the scope of this review.

**Figure 21:**
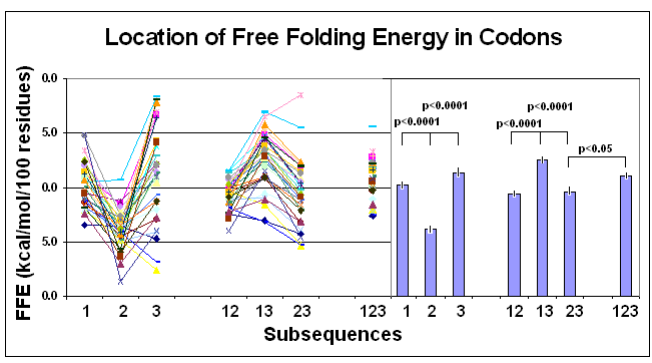
Location of free folding energy in codons. Free folding energies (FFE) were determined in phase-selected subsequences of 31 different protein-coding nucleic acids. The original intact RNA contained the intact three-letter codons (123). Subsequences were constructed by periodical removal of one letter from the codon while maintaining the other two (12, 13, 23) or removing two letters and maintaining only one (1, 2, 3). The lines indicate individual values (left), while the bars (right) indicate the mean ± SEM (*n *= 31).

That the FFE in subsequences of 1st and 3rd codon residues is higher than in the 2nd indicates the presence of a larger number of complementary bases at the right positions of these subsequences. However, this might be the case only because the first and last codons form simpler subsequences and contain longer repeats of the same nucleotide than the 2nd codons. This would not be surprising for the 3rd (wobble) base but would not be expected for the 1st residue, even though the central codon letters are known to be the most important for distinguishing between amino acids (as shown in the Common Periodic Table of Codons and Amino Acids [52]. It is more significant that the FFEs in 1st and 3rd residues are additive and together they represent the entire FFE of the intact mRNA (Figure 21).

That the FFE at the 1st and 3rd codon positions is higher than at 2nd also indicates that the number of complementary bases (a-t and g-t) is higher in the 1st and 3rd subsequences than in the second. This is possible only if more complementers are in 1-1, 1-3, 3-1, 3-3 position pairs than in 1-2, 2-1, 2-3, 3-2 position pairs. We wanted to know whether the 1-1, 3-3 (complement) or the 1-3, 3-1 (reverse-complement) pairing is more predominant.

The length of phase-separated nucleic acid subsequences (*l*) is a third of the original coding sequence (*L*). The number of different residues (a, t, g, and c) varies at different codon positions (1, 2, 3).

a1 + u1 + g1 + c1 = a2 + t2 + g2 + c2 = a3 + t3 + g3 + c3 = *l *= *L*/3

The highest number of complementary pairs might occur in the 1st subsequence if

a1 = t1, g1 = c1 and a1/t1 = g1/c1 = 1

**Figure 22:**
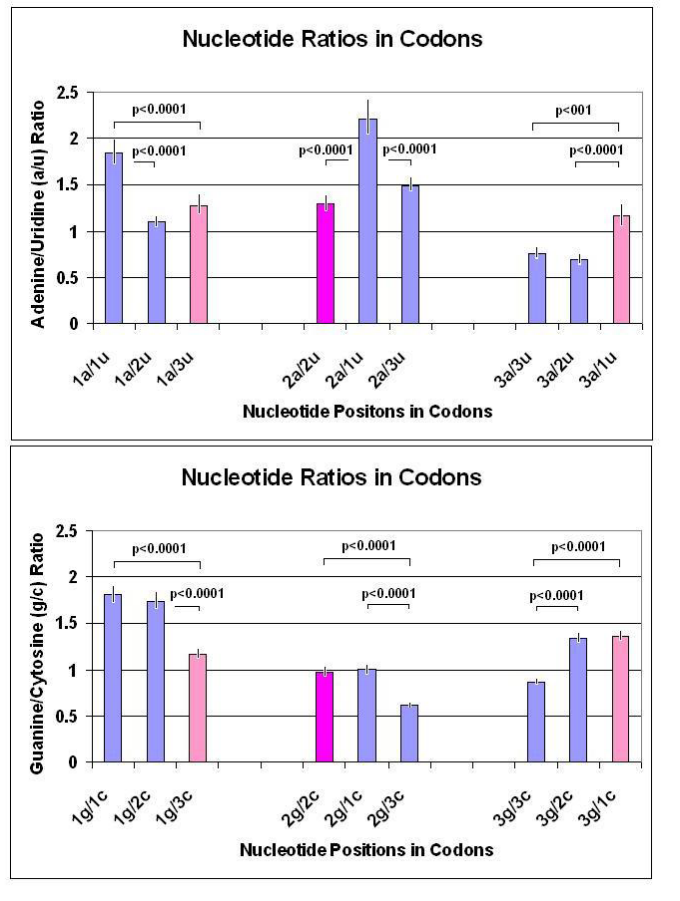
Nucleotide ratios in codons. The number of the 4 different nucleotide bases was counted at the 1st, 2nd and 3rd codon positions in 30 different protein coding RNA sequences. The ratios of the Watson-Crick pairs at different codon positions are indicated by bars (± SEM, *n *= 30). Ideally, the ratio of complementary base pairs is ~1.0. This ideal situation was mostly satisfied when one of the complementary bases was located at codon position 1 with the other at codon position 3 (pink) or both complements at codon position 2 (violet).

If, for example, a1 > t1, g1 = c1 an excess of unpaired a1 occurs and a1/t1 > g1/c1 = 1 and the possible FFE in subsequence 1 will be lower. Following the same logic for other pairs in other subsequences we can conclude that any deviation from a/t = g/c = 1 is suboptimal regarding the FFE. Counting the different residue ratios and combinations indicates that the optima are obtained if the residues in the first position form W-C pairs with residues at the third positions (1-3) and vice versa (3-1). This is consistent with the expectation that mRNA will form local loops, in which the direction of more or less double stranded sequences is reversed and (partially) complemented (Figure 22).

### Comparison of the protein and mRNA secondary structures

**Figure 23:**
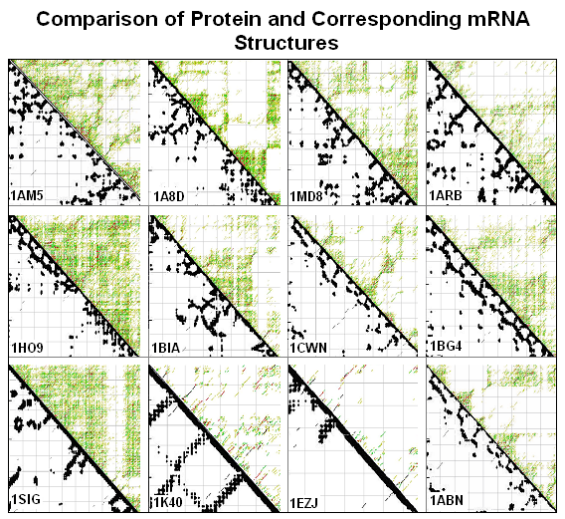
Comparison of protein and corresponding mRNA structures (modified from [95]). Residue contact maps (RCM) were obtained from the PBD files of protein structures using the SeqX tool (left triangles). Energy dot-plots (EDP) for the coding sequences were obtained using the mfold tool (right triangles). The two kinds of maps were aligned along a common left diagonal axis to facilitate visual comparison of the different kinds of representation possible. The black dots in the RCMs indicate amino acids that are within 6 A of each other in the protein structure. The colored (grass-like) areas in the EDPs indicate the energetically mostly likely RNA interactions (color code in increasing order: yellow, green red, black).

The partial (suboptimal) reverse complementarity of codon-related positions in nucleic acids suggested some similarity between protein structures and the possible structures of the coding sequences. This suggestion was examined by visual comparison of 16 randomly selected protein residue contact maps and the energy dot-plots of the corresponding RNAs. We could see similarities between the two different kinds of maps (Figure 23). However, this type of comparison is not quantitative and statistical evaluation is not directly possible.

**Figure 24:**
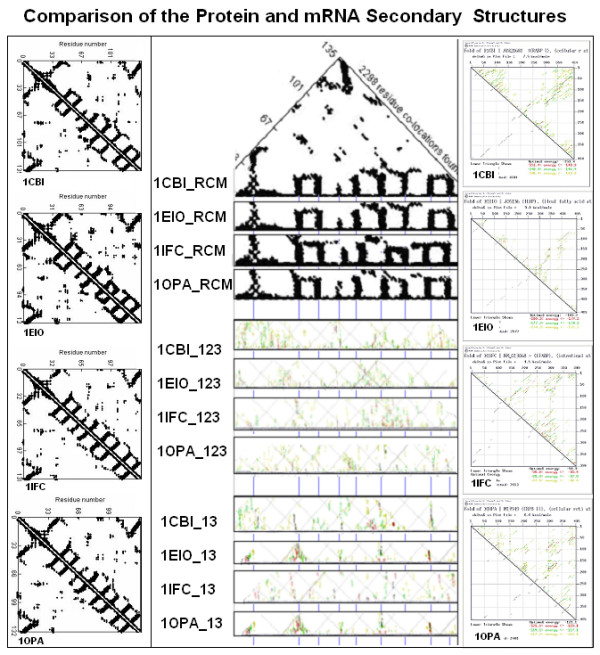
Comparison of the protein and mRNA secondary structures (modified from [95]). Residue contact maps (RCM) were obtained from the PBD files of 4 protein structures (1CBI, 1EIO, 1IFC, 1OPA) using the SeqX tool (left column). Energy dot-plots (EDP) for the coding sequences were obtained using the mfold tool (right column). The left diagonal portion of these two kinds of maps was compared in the central part of the figure. Blue horizontal lines in the background correspond to the main amino acid co-location sites in the RCM. Intact RNA (123) and subsequences containing only the 1st and 3rd codon letters (13) are compared. The black dots in the RCMs indicate amino acids that are within 6 A of each other in the protein structure. The colored (grass-like) areas in the EDPs indicate the energetically most likely RNA interactions (color code in increasing order: yellow, green red, black).

Another similar, but still not quantitative, comparison of protein and coding structures was performed on four proteins that are known to have very similar 3D structures but their primary structures (sequences) and the sequences of their mRNAs are less than 30% similar. These four proteins exemplify the fact that the tertiary structures are much more conserved than amino acid sequences. We asked whether this is also true for the RNA structures and sequences. We found that there are signs of conservation of the RNA secondary structure (as indicated by the energy dot-plots) and there are similarities between the protein and nucleic acid structures (Figure 24).

**Figure 25:**
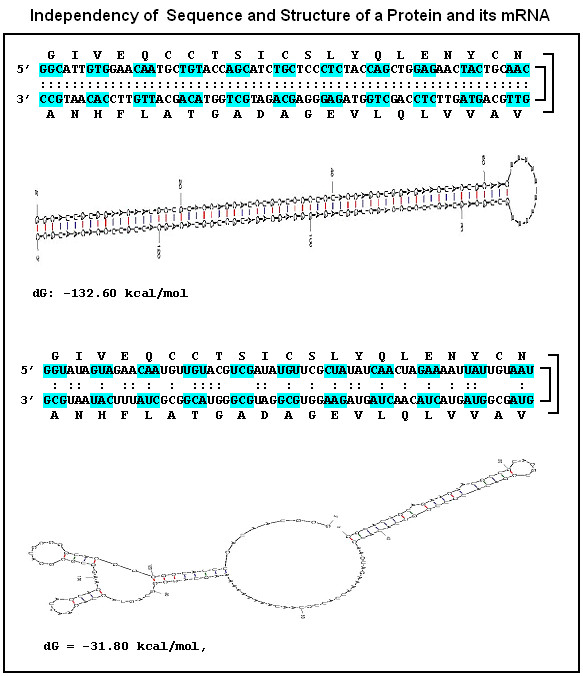
Independence of sequence and structure of a protein and its mRNA. The amino acids in a U-shaped protein structure are encoded by complementary codons (rule PC1). The nucleic acid structure is uniform and the folding energy is -132 kcal/mol. Exactly the same amino acid sequence might be encoded by non- or only partially complementary codons, which will fundamentally alter the mRNA structure and increase the folding energy to -31 kcal/mol (less stable). The nucleic acid structures were generated by mfold and dGs were calculated by the same program.

The similarity between mRNA and the encoded protein secondary structures is an unexpected, novel observation. The 21/64 redundancy of the genetic code gives a 441/4.096 codon pair redundancy for every amino acid pair. It means that every amino acid pair might be coded by ~9 different codon pairs (some are complementary but most are not). The similarity between protein and corresponding mRNA structures indicates extensive complementary coding of co-locating amino acids. The possible number of codon variations and possible nucleic acid structures behind a protein sequence and structure is very large (Figure 25) and the same applies to the corresponding folding energies (d*G*, the stability of the mRNA).

### Complementary codes vs amino acid co-locations

Comparisons of the protein residue contact map with the nucleic acid folding maps suggest similarities between the 3D structures of these different kinds of molecules. However, this is a semi-quantitative method.

More direct statistical support might be obtained by analyzing and comparing residue co-locations in these structures. Assume that the structural unit of mRNA is a tri-nucleotide (codon) and the structural unit of the protein is the amino acid. The codon may form a secondary structure by interacting with other codons according to the W-C base complementary rules, and contribute to the formation of a local double helix. The 5'-A1U2G3-3' sequence (Met, M codon) forms a perfect double string with the 3'-U3A2C1-5' sequence (His, H codon, reverse and complementary reading). Suboptimal complexes are 5'-A1X2G3-3' partially complemented by 3'-U3X2C1-5' (AAG, Lys; AUG, Met; AGG, Arg; ACG, Pro; and CAU, His; CUU, Leu; CGU, Arg; CCU, Pro, respectively).

**Figure 26:**
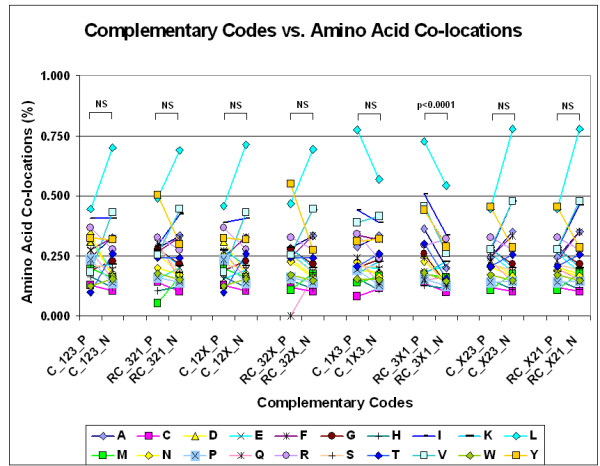
Complementary codes vs amino acid co-locations (modified from [95]). The propensities for the 400 possible amino acid pairs were monitored in 81 different protein structures with the SeqX tool. The tool detected co-locations when two amino acids were within 6 A of each other (neighbors on the same strand were excluded). The total number of co-locations was 34,630. Eight different complementary codes were constructed for the codons (2 optimal and 6 suboptimal). In the two optimal codes, all three codon residues (123) were complementary (C) or reverse complementary (RC) to each other. In the suboptimal codes, only two of three codon residues were C or RC to each other (12, 13, 23), while the third was not necessarily complementary (X). (For example, Complementary Code RC_1X3 means that the first and third codon letters are always complementary, but the not the second, and the possible codons are read in reverse orientation. The 400 co-locations were divided into 20 subgroups corresponding to 20 amino acids (one of the co-locating pairs), each group containing the 20 amino acids (corresponding to the other amino acid in the co-locating pair). If the codons of the amino acid pairs followed the predefined complementary code the co-location was regarded as positive (P); if not, the co-location was regarded as negative (N). Each symbol represents the mean frequency of P or N co-locations corresponding to the indicated amino acid. Paired Student's *t*-test, *n *= 20.

**Figure 27:**
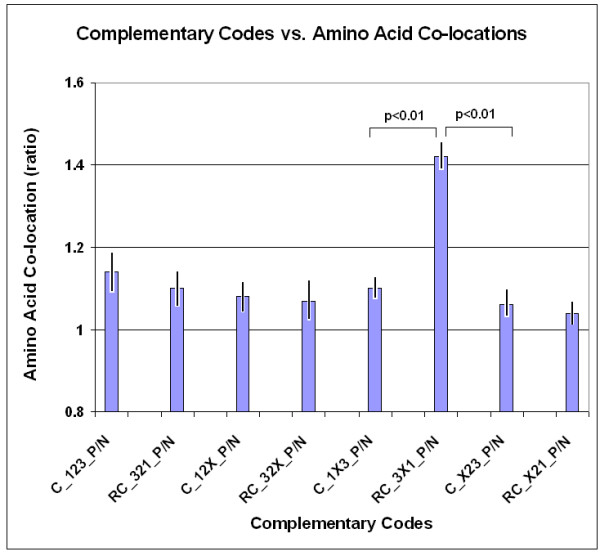
Complementary codes vs amino acid co-locations (ratios) (modified from [95]). The ratio of positive (P) and negative (N) co-locations was calculated on data from Figure 13. Each bar represents the mean ± SEM (*n *= 20).

Our experiments with FFE indicate that local nucleic acid structures are formed under this suboptimal condition, i.e., when the 1st and 3rd codon residues are complementary but the 2nd is not. If this is the case, and there is a connection between nucleic acid and protein 3D structures, one might expect that the 4 amino acids encoded by 5'-A1X2G3-3' codons will preferentially co-locate with the 4 different amino acids encoded by 3'-U3X2C1-5' codons. We constructed 8 different complementary codon combinations and found that the codons of co-locating amino acids are often complementary at the 1st and 3rd positions and follow the D-1X3/RC-3X1 formula but not the other seven formulae (Figures 26 and 27).

**Figure 28:**
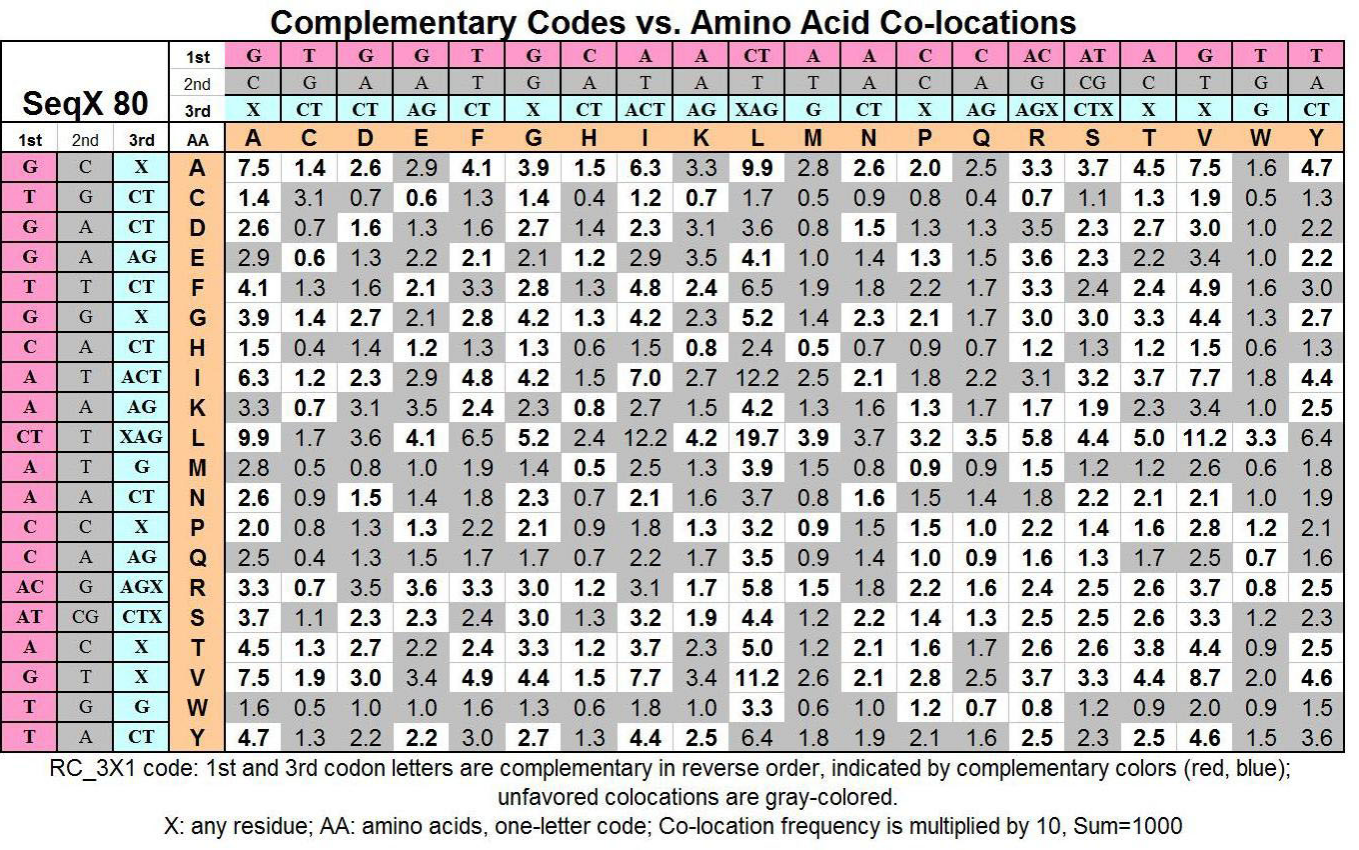
Complementary codes vs amino acid co-locations. See text for explanation.

These special amino acid pairs and their frequencies are indicated and summarized in a matrix (Figure 28).

It is well known that coding and non-coding DNA sequences (exon/intron) are different and this difference is somehow related to the asymmetry of the codons, i.e., that the third codon letter (wobble) is poorly defined. Many Markov models have been formulated to find this asymmetry and predict coding sequences (genes) de novo. These in silico methods work rather well but not perfectly and some scientists remain unconvinced that codon asymmetry explains the exon-intron differences satisfactorily.

Another codon-related problem is that the well-known, non-overlapping triplet codon translation is extremely phase-dependent and there is theoretically no tolerance of any phase shift. There are famous examples of how a single nucleotide deletion might destroy the meaningful translation of a sequence and are incompatible with life. However, considering the magnitude and complexity of the eukaryotic proteome, the precision of translation is astonishingly good. Such physical precision is not possible without a massive and consistent physico-chemical basis. Therefore, discovery of the existence of secondary structure bias (folding energy differences) in coding regions of many organisms [77] was a very welcome observation because it differentiates exons from introns physico-chemically.

Our experiments with free folding energy (FFE) confirmed that this bias exists. In addition, there is a very consistent and very significant pattern of FFE distribution along the nucleotide sequence. Comparing the FFE of phase-selected subsequences, subsequences comprising only the 1st or only the 3rd codon letters showed significantly higher FFE than those consisting only of the 2nd letters. This FFE difference was not present in intronic sequences preceding and following the exons, but it was present in exons from different species including viruses. This is an interesting observation because this phenomenon might not only distinguish between exons and introns on a physico-chemical basis, but might also clearly define the tri-nucleotide codons and thus the phase of the translation. This codon-related phase-specific variation in FFE may explain why mRNAs have greater negative free folding energies than shuffled or codon choice randomized sequences [84].

Free folding energy in nucleic acids is always associated with W-C base pair formation. Higher FFE indicates more W-C pairs (presence of complementarity) and lower FFE indicates fewer W-C pairs (less complementarity). The FFE in the 1st and 3rd codon positions was additive, while the 2nd letter did not contribute to the total FFE; the total FFE of the entire (intact) nucleic acid was the same as subsequences containing only the 1st and 3rd codon letters (2nd deleted). This is an indication that the local RNA secondary structure bias is caused by complementarity of the 1st and 3rd codon residues in local sequences. This partial, local complementarity is more optimal in reverse orientation of the local sequences, as expected with loop formation.

It is known that single stranded RNA molecules can form local secondary structures through the interactions of complementary segments. The novel observation here is that these interactions preferentially involve the 1st and 3rd codon residues. This connection between RNA secondary structure and codons immediately directed attention towards the question of protein folding and its long-suspected connection to RNA folding [85,86].

Only about one-third (20/64) of the genetic code is used for protein coding, i.e., there is a great excess of information in the mRNA. At the same time, the information carried by amino acids seems to be insufficient (according to some scientists) to complete unambiguous protein folding. Therefore, it is believed that the third codon residue (wobble base) carries some additional information to that already present in the genetic code. A specialized wobble base-oriented database, the ISSD [83], was established in an effort to connect different features of protein structure to wobble bases [87] more or less successfully.

We found a significant negative correlation between FFE of the 2nd codon residue and the helix content of protein structures, which was not expected even though this possibility is mentioned in the literature [73]. Our previous work on a Common Periodic Table of Codons and Nucleic Acids [52] indicated that the second codon residue is intimately coupled with the known physico-chemical properties of the amino acids. Almost all amino acids show significant positive or negative correlation to the helix content of proteins. Therefore, the real biological meaning and significance of any connection between the FFE of the 2nd codon residue and the propensity towards a protein structural element is highly questionable.

A working hypotheses grew out of these observations, namely that (a) partial, local reverse complementarity exists in nucleic acids and forms the nucleic acid structure; (b) there is some degree of similarity between the folding of nucleic acids and proteins; (c) nucleic acid structure determines the amino acid co-locations; (d) as a consequence, amino acids encoded by the interacting (partially reverse complementary) codons might show preferential co-locations in the protein structures.

This seems to be the case: codons that contain complementary bases at the 1st and 3rd positions and are translated in reverse orientation result in amino acids that are preferentially co-located (interacting) in the 3D protein structure. Other complementary residue combinations or translation in the same (not reverse) direction (as much as seven combinations in total) did not result in any preferentially co-locating subset of amino acid pairs.

Construction of residue contact maps for protein structures and statistical evaluation of residue co-locations is a frequently used method for visualizing and analyzing spatial connections between amino acids [88-90]. The amino acid co-locations in real protein structures are clearly not random [91,92] and therefore residue co-location matrices are often used to assist in the prediction of novel protein structures [93,94]. We have carefully examined the physico-chemical properties of specifically interacting amino acids in and between protein structures, and we concluded that these interactions follow the well-known physico-chemical rules of size, charge and hydrophobe compatibility (unpublished data), well in line with Anfinsen's prediction. A recent study supports the view that there is a previously unknown connection between the codons of specifically interacting amino acids; those codons are complementary at the 1st and 3rd (but not the 2nd) codon positions.

The idea that sequence complementarity might explain the nature of specific protein-protein interactions is not new and was suggested as early as 1981 [9-11].

I was never able to confirm my own original theory(perfect complementarity between codons of interacting amino acids [9-11,50] experimentally, in contrast to others [37]. The explanation is that this codon complementarity is suboptimal and does not involve the 2nd codon residue. Experimental in vitro confirmation is required to validate this recent theoretical and in silico prediction.

### Theory of nucleic acid (chaperon)-assisted protein folding

**Figure 29:**
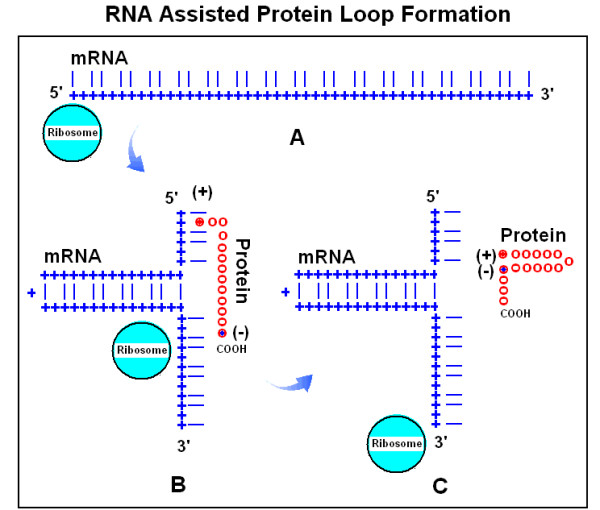
RNA-assisted protein loop formation (from [95]). Translation begins with the attachment of the 5' end of a mRNA to the ribosome (A). Ribonucleotides are indicated by blue "+" and the 1st and 3rd bases in the codons by blue lines; the 2nd base positions are left empty. A positively charged amino acid ((+) and red dots), for example arginine, remains attached to its codon. The mRNA forms a loop because the 1st and 3rd bases are locally complementary to each other in reverse orientation (B). The growing protein is indicated by red circles. When translation proceeds to an amino acid with especially high affinity for the mRNA-attached arginine, for example a negatively charged Glu or Asp ((-) and blue dot), the charge attraction removes the Arg from its mRNA binding site and the entire protein is released from the mRNA and completes a protein loop (C). The protein continues to grow towards the direction of its carboxy terminal (COOH).

**Figure 30:**
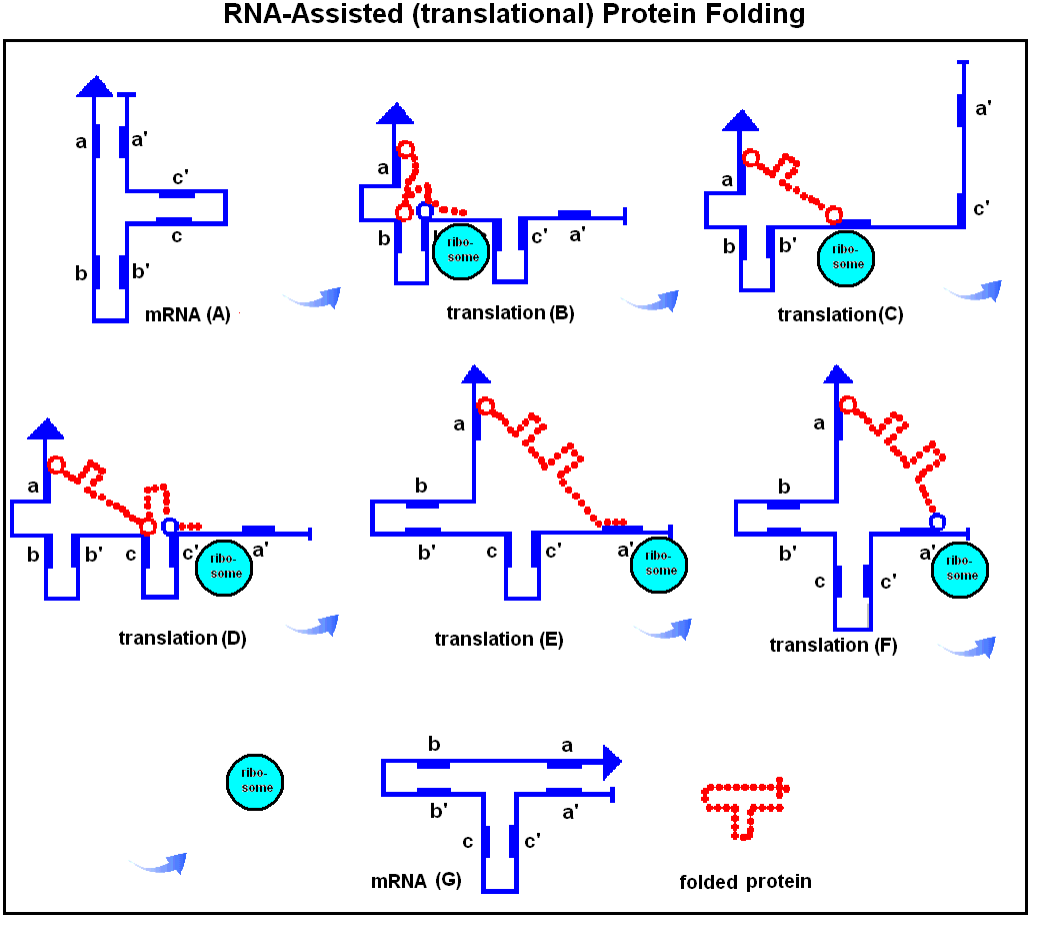
RNA-assisted (translational) protein folding (from [95]. There are three reverse and complementary regions in a mRNA (blue line, A): a-a', b-b', c-c', which fold the mRNA into a T-like shape. During the translation process the mRNA unfolds on the surface of the ribosome, but subsequently refolds, accompanied by its translated and lengthening peptide (red dotted line, B-F). The result of translation is a temporary ribonucleotide complex, which dissociates into two T-shape-like structures: the original mRNA and the properly folded protein product (G). The red circles indicate the specific, temporary attachment points between the RNA and protein (for example a basic amino acid); the blue circles indicate amino acids with exceptionally high affinity for the attachment points (e.g., acidic amino acids); these capture the amino acids at the attachment point and dissociate the ribonucleoprotein complex. Transfer RNAs are of course important participants in translation, but they are not included in this scenario.

So far, a series of novel arguments has been presented to support a deeper connection than the traditional codon translation between nucleic acids and expressed proteins. The physico-chemical properties of amino acids are clearly associated with the 2nd codon letter as shown in the Common Periodic Table of Codons and Amino Acids. The co-locating amino acids are preferentially encoded by codons that are complementary at the 1st and 3rd positions. The structures of proteins and their encoding nucleic acids are rather similar to each other in many cases. All these observations suggest the co-evolution of codons and amino acids and that protein folding (structural) information is present in the nucleic acids in addition to the canonical genetic code. This immediately raises the possibility of nucleic acid-assisted protein folding, i.e. the possibility of nucleic acid chaperons [95]. This is an exciting possibility, because the protein primary sequence seems not to carry all the necessary information for unambiguous protein folding (in conflict with Anfinsen's theorem), while there is a twofold excess of information in the redundant genetic code. A theoretical example of how such nucleic acid-assisted protein folding may look is presented in Figures 29 and 30.

## Definition of the 2nd generation Proteomic Code

The Proteomic Code is a set of comprehensive rules by which information in genetic material is transferred into the physico-chemical properties of amino acids and determines how individual amino acids interact with each other during protein folding and in specific protein-protein interactions. The Proteomic Code is part of the redundant genetic code. The theory of Proteomic Code contains the following observations:

- Co-locating (interacting) amino acids in native proteins are encoded by partially (imperfect) complementary codons in reverse (5'→3'/5'→3') orientation.

- Partial complementarity means that the 1st and 3rd codon bases are complementary (Watson-Crick) bases to each other, while the 2nd bases may or may not be complementary to each other.

- The physico-chemical characteristics of the coded and interacting amino acids are determined mainly by the 2nd (central) codon residues.

- The physico-chemical properties of the interacting amino acids (size, charge, and hydropathy) are compatible with each other at the individual amino acid level.

- Nucleic acids (exons) contain protein folding information within (or in addition to) the redundant genetic code.

- Nucleic acids may directly assist protein folding as chaperons.

**Figure 31:**
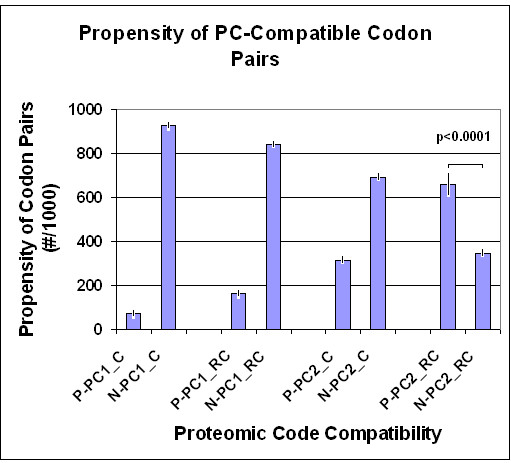
Propensity for PC-compatible codon pairs. There are 4096 possible codon pairs altogether (64 × 64, including those formed with the 3 stop codons). Codons that are (P) or are not (N) coded by a specific complementary rule (PC) were counted. The bars represent the mean ± SD of 9 independent determinations (*n *= 9). PC1 and PC2 indicate complementarity of all 3 or only the first and third codon bases in parallel (C) or anti-parallel (RC) readings.

**Figure 32:**
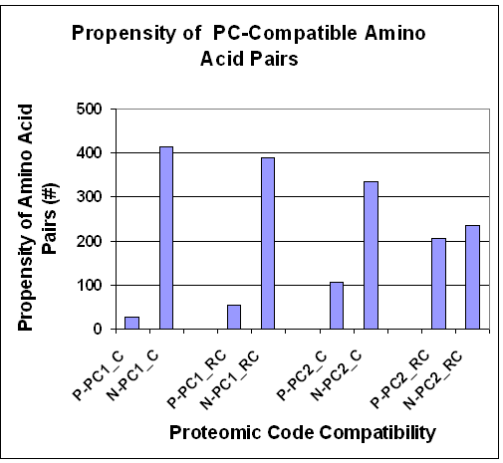
Propensity for PC-compatible amino acid pairs. There are 441 possible amino acid pairs altogether (21 × 21, including the stop place as the 21st variable). Amino acids that are (P) or are not (N) coded by a specific complementary rule (Proteomic Code, PC) were counted. PC1 and PC2 indicate complementarity of the respective codon pairs at all 3 or only the first and third codon bases in parallel (C) or anti-parallel (RC) readings.

There are four different Proteomic Codes at this moment. PC1_C and PC1_RC are the original codes based on the perfect complementarity of all three codon bases in complementary (C, 5'→3'/3'→5') readings; PC2_C and PC2_RC are the recently extended codes requiring base pair complementarity at the 1st and 3rd codon positions but not necessarily at the 2nd. PC1 is part of PC2. The PC_C variants require 3'→5' translations, which do not exist, therefore I regard this variant as an artifact, caused by the symmetry of many codons. Only a small percentage codon and amino acid pairs belongs to PC1; ~50% of all amino acid pairs and >60% of all codon pairs can be classified into PC2_RC (Figures 31 and 32).

All possible amino acid pairs (21 × 21 = 441, including the virtual pairs formed with the Stop/End signal), are listed in a Table [see Additional file 2]. The most important physico-chemical parameters (molecular weight (MW), isoelectric point (p*I*), hydropathy (HP)) and the derived values of three compatibility indexes (charge (CCI), size (SCI) and hydropathy (HCI) compatibility indices [58]) as well as the expected frequency of the amino acid pairs (natural frequency (NF), calculated from codon table 1) are included in Table 4. P (positive) and N (negative) indicate whether an amino acid pair may or may not be coded by a given Proteomic Code.

### The Origin of Proteomic Code as part of the Genetic Code

The theory of Ikehara [reviewed in [96,97]] about the origins of gene, genetic code, protein and life is especially interesting regarding the Proteomic Code. Ikehara suggests (and support with experimental evidence) that gene-protein system, comprised of 64 codons and 20 amino acids developed successfully during the evolution.

The development started with a *GNC-type primeval genetic code *(G: guanine, C: Cytosine, N: any of the four nucleotides), coding only four amino acids (Gly: [G], Ala: [A], Asp: [D], Val: [V]) forming the so called [GADV]-proteins. This minimal set of only four amino acids and the [GADV]-proteins are able to represent the 6 major (and characteristic) protein moieties/indices (hydropathy, *a*-helix, *b*-sheet and *b*-turn forms, acidic amino acid content and basic amino acid content) which are necessary for appropriate three-dimensional structure formation of globular, water-soluble proteins on the primitive earth. The [GADV]-proteins (even randomized) have catalytic properties and able to facilitate the syntheses of other [GADV]-proteins (also random).

The primeval genetic code continued to develop toward a more complex *SNS-type primitive genetic code *(S: G or C) containing 16 codons and encoding 10 amino acids (L, P, H, Q, R, V, A, D, E, G) before the recent *64 codon/20 amino acid-type recent genetic code *became established.

Furthermore, Ikehara concluded from the analysis of microbial genes that newly-born genes are products of nonstop frames (NSF) on antisense strands of microbial GC-rich genes [GC-NSF(antisense)] and from SNS repeating sequences [(SNS)n] similar to the GC-NSF(antisense).

The similarity between GNC/SNS-type primitive codons (which are expressed even from the reverse-complement strands as GC-rich non-stop genes) and the Proteomic Code is obvious. Both concepts suggest and agree with each other regarding

a) the connection between 2^nd ^codon residue and the fundamental physicochemical properties of the coded amino acids,

b) the importance of 1^st ^and 3^rd ^codon letters in determining the nucleic acid (as well as protein) structure,

c) the importance of compositional difference between 1^st^, 3^rd ^and central codon residues (to emphasize the codon boundaries),

d) the importance of complementarity (even in the mRNA) in development of protein structure and function,

e) the importance of GC at the 1^st ^and 3^rd ^codon positions (as the source of lower Gibbs energy, than central codon positions have, where even AT are permitted).

I think, that the concept of GNC/SNS-type primitive codons and the Proteomic Code are convergent ideas, both reflecting the same fundamental aspects of the connection between nucleic acid and protein structure and function.

## System and method for obtaining oligo-peptides with specific high affinity for query proteins

**Figure 33:**
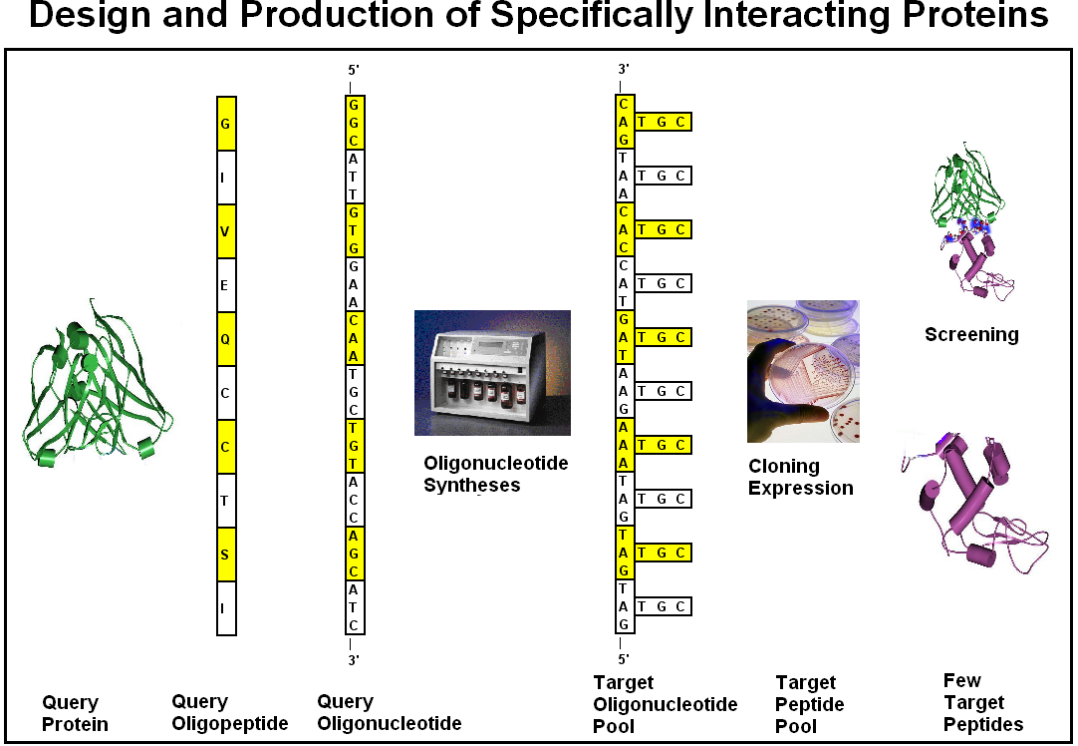
Design and production of specifically interacting proteins (see text for details).

**Figure 34:**
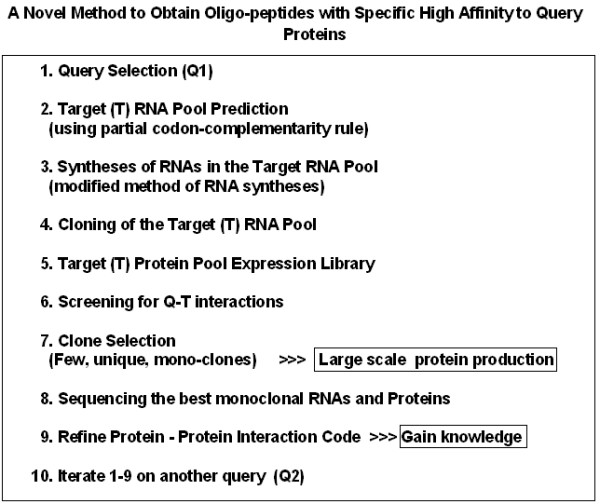
Steps constituting the novel method for obtaining oligo-peptides with specific high affinity for query proteins.

I have developed a system and method for obtaining oligo-peptides with specific high affinity for query proteins [98]. The method is based on the second generation Proteomic Code. Figures 33 and 34 show the steps in this system for producing the target proteins with a high affinity for query proteins; it is assumed the primary structures of the query proteins are known.

There is no limitation to the size of the query; however, the sequence is preferably in the range 5–40 amino acid residues, and best in the range 7–15. Preferably, the real and natural coding sequence is known for the query protein. However, there might be some special cases in which the sequence is not exactly known, for example designed or artificially modified proteins. Thus, it is possible to fabricate a virtual coding sequence with back translation using Codon Usage Frequency Tables. The present method relies on the entire information carried by the naturally-occurring DNA/mRNA and not only that used for coding of the protein primary sequence.

The query sequence should be a "promising" domain of the query protein and specific domains are more important, including domains that: (a) are known to be antigenic; (b) are located on the surface of the query protein; (c) are not simple (repetitive) sequences; (d) contain less frequent amino acids; and (e) contain charged amino acid residues.

Once the promising area of the known amino acid sequence is chosen and the nucleotide sequence is determined, then construction of nucleic acid sequences encoding the target proteins is initiated. The target nucleotide (RNA or DNA) prediction should follow a simple rule, namely that the 1st and 3rd codon letters of the target nucleotide sequences should be reverse-complementary to the 1st and 3rd codon nucleotide residues of the query nucleotide sequence, but the middle, 2nd residue can be any of the four possible nucleotides. The expected number of predicted target RNAs will be 4^*n*^, where *n *is the number of amino acids (= number of codons, = number of 2nd codon letters).

Nucleotide sequences can be produced readily, for example, directly synthesizing the fragment by chemical means, applying nucleic acid reproduction technology such as PCR, or excision of selected DNA fragments from recombinant plasmids containing appropriate inserts and suitable restriction enzyme sites. However, synthesis of predicted (max. 4^n^) sequences on a one by one basis does not seem practical. Thus, a simple mass-production method is needed that will result in a mixture containing all possible sequences in the predicted RNA/DNA pool. Fortunately, the regular nature of the nucleotides in the pool makes it possible to synthesize the entire pool of sequences as if it were only one single nucleotide sequence. For example, the usual step-by-step (base by base) protocol can be followed except at the positions for the synthesis of the 2nd codon residue. At those points in the synthesis process, an equal mixture of the four nucleotides should be provided instead of a single nucleotide. The result of this modified oligo-nucleotide synthesis should be a mixture of the desired potential target RNAs.

The predicted and synthesized RNAs in the pool are cloned by the standard procedure, which involves insertion of RNA into vector (plasmid or other carrier) and multiplying the sequences in bacteria or yeast as described in the literature. Expression vectors of the system may comprise polynucleotides operatively linked to an enhancer-promoter such as a prokaryotic or eukaryotic promoter. Further, an enhancer may be included in the vector. A major function of an enhancer is to increase the level of transcription of a coding sequence in a cell that contains one or more transcription factors that bind to that enhancer. Unlike a promoter, an enhancer can function when located at variable distances from transcription start sites so long as a promoter is present.

Expression vectors of the present system comprise polynucleotides that encode the target peptides of the pool. Where expression of recombinant polypeptide is desired and a eukaryotic host is contemplated, it is best to employ a vector such as a plasmid that incorporates a eukaryotic origin of replication. In addition, for the purposes of expression in eukaryotic systems, it is desired to position the peptide-encoding sequence adjacent to and under the control of an effective eukaryotic promoter such as those used in combination with Chinese hamster ovary cells. To bring a coding sequence under the control of a promoter, whether it is eukaryotic or prokaryotic, what is generally needed is to position the 5' end of the translation initiation side of the proper translational reading frame of the polypeptide between about 1 and 50 nucleotides 3' of (or downstream with respect to) the promoter chosen. Furthermore, where eukaryotic expression is anticipated, one would typically desire to incorporate an appropriate polyadenylation site into the transcriptional unit that includes the different target peptides.

pRc/CMV (available from Invitrogen) is an exemplary vector for expressing a peptide in mammalian cells, particularly COS and CHO cells. Target polypeptides of the present invention under the control of a CMV promoter can be efficiently expressed in mammalian cells. The pCMV plasmids are a series of mammalian expression vectors of particular utility in the present system. The vectors are designed for use in essentially all cultured cells and work extremely well in SV40-transformed simian COS cell lines. The pCMV1, 2, 3 and 5 vectors differ from each other in certain unique restriction sites in the polylinker region of each plasmid. The pCMV4 vector differs from these 4 plasmids in containing a translation enhancer in the sequence prior to the polylinker. While they are not directly derived from the pCMV1-5 series of vectors, the functionally similar pCMV6b and c vectors, available from the Chiron Corp. (Emeryville, CA), are identical except for the orientation of the polylinker region, which is reversed in one relative to the other. The pCMV vectors have been successfully expressed in simian COS cells, mouse L cells, CHO cells and HeLa cells.

Means of transforming or transfecting cells with exogenous polynucleotide such as the nucleotide molecules of the present system are well known and include techniques such as calcium-phosphate- or DEAE-dextran-mediated transfection, protoplast fusion, electroporation, liposome-mediated transfection, direct microinjection and adenovirus infection. The most widely-used method is transfection mediated by either calcium phosphate or DEAE-dextran. Although the mechanism remains obscure, it is believed that the transfected DNA enters the cytoplasm by endocytosis and is transported to the nucleus. Depending on the cell type, up to 90% of a population of cultured cells can be transfected at any one time. Because of its high efficiency, transfection mediated by calcium phosphate or DEAE-dextran is the method of choice for experiments that require transient expression of the foreign DNA in large numbers of cells. Calcium phosphate-mediated transfection is also used to establish cell lines that integrate copies of the foreign DNA, which are usually arranged in head-to-tail tandem arrays into the host cell genome.

The application of brief, high-voltage electric pulses to a variety of mammalian and plant cells leads to the formation of nanometer-sized pores in the plasma membrane. DNA is taken directly into the cytoplasm either through these pores or as a consequence of the redistribution of membrane components that accompanies closure of the pores. Electroporation can be extremely efficient and can be used both for transient expression of cloned genes and for establishment of cell lines that carry integrated copies of the gene of interest. Electroporation, in contrast to calcium phosphate-mediated transfection and protoplast fusion, frequently gives rise to cell lines that carry one, or at most a few, integrated copies of the foreign DNA.

Liposome transfection involves encapsulation of DNA or RNA within liposomes, followed by fusion of the liposomes with the cell membrane. The mechanism by which DNA or RNA is delivered into the cell is unclear but transfection efficiencies can be as high as 90%.

Direct microinjection of a DNA molecule into the nucleus has the advantage of not exposing DNA to cellular compartments such as low pH endosomes. Microinjection is therefore used primarily as a method for establishing lines of cells that carry integrated copies of the DNA of interest. A transfected cell can be prokaryotic or eukaryotic.

In addition to prokaryotes, eukaryotic microbes such as yeast can also be used. *Saccharomyces cerevisiae *is the most commonly used eukaryotic microorganism, although a number of other strains are also available. For expression in *Saccharomyces*, the plasmid YRp7, for example, is commonly used. This plasmid already contains the trpl gene, which provides a selection marker for a mutant strain of yeast lacking the ability to grow in tryptophan, for example ATCC No. 44076 or PEP4-1. The presence of the trpl lesion as a characteristic of the yeast host cell genome then provides an effective environment for detecting transformation by growth in the absence of tryptophan. Suitable promoter sequences in yeast vectors include the promoters for 3-phosphoglycerate kinase or other glycolytic enzymes such as enolase, glyceraldehyde-3-phosphate dehydrogenase, hexokinase, pyruvate decarboxylase, phosphofructokinase, glucose-6-phosphate isomerase, 3-phosphoglycerate mutase, pyruvate kinase, triosephosphate isomerase, phosphoglucose isomerase and glucokinase. In constructing suitable expression plasmids, the termination sequences associated with these genes are also introduced into the expression vector downstream from the sequences to be expressed to provide polyadenylation of the mRNA and termination. Other promoters, which have the additional advantage of transcription controlled by growth conditions, are those for alcohol dehydrogenase 2, isocytochrome *c*, acid phosphatase, degradative enzymes associated with nitrogen metabolism, the aforementioned glyceraldehyde-3-phosphate dehydrogenase, and enzymes responsible for maltose and galactose utilization. Any plasmid vector containing a yeast-compatible promoter, origin or replication and termination sequences is suitable.

In addition to microorganisms, cultures of cells derived from multicellular organisms can also be used as hosts. In principle, any such cell culture is workable, whether from vertebrate or invertebrate culture. However, interest has been greatest in vertebrate cells, and propagation of vertebrate cells in culture (tissue culture) has become a routine procedure in recent years. Examples of such useful host cell lines are AtT-20, VERO and HeLa cells, Chinese hamster ovary (CHO), and W138, BHK, COSM6, COS-1, COS-7, 293 and MDCK. Expression vectors for such cells ordinarily include (if necessary) an origin of replication, a promoter located upstream of the gene to be expressed, along with any necessary ribosome binding sites, RNA splice sites, polyadenylation site, and transcriptional terminator sequences.

For use in mammalian cells, the control functions on the expression vectors are often derived from viral material. For example, commonly used promoters are derived from polyoma, Adenovirus 2, Cytomegalovirus and most frequently Simian Virus 40 (SV40). The early and late promoters of SV40 are particularly useful because both are obtained easily from the virus as a fragment that also contains the SV40 origin of replication. Smaller or larger SV40 fragments can also be used, provided they include the approximately 250 bp sequence extending from the HindIII site towards the BglI site located in the viral origin of replication. It is also possible, and often desirable, to utilize promoter or control sequences normally associated with the desired gene sequence, provided such control sequences are compatible with the host cell systems.

Culture conditions are well known and include ionic composition and concentration, temperature, pH, etc. Typically, transfected cells are maintained under culture conditions; suitable media for various cell types are well known. Temperature is preferably from about 20°C to about 50°C. pH is preferably from about 6.0 to about 8.0, better in the range 6.8–7.8 and best at about 7.4. Other biological conditions needed for transfection and expression of an encoded protein are well known.

Following transfection, the cell is maintained under culture conditions for a period of time sufficient for the target proteins of the pool to be expressed. A suitable time depends inter alia upon the cell type used and is readily determinable by a skilled technician. Typically, maintenance time is from about 2 to 14 days. Recovery of the target proteins comprises isolating and purifying the recombinant polypeptides. Isolation and purification techniques for polypeptides are well known and include such procedures as precipitation, filtration, chromatography, electrophoresis, etc.

The target proteins are preferably arranged in a library assay system for screening with samples of the query protein. Any method that detects specific, high affinity protein-protein interactions is theoretically useful for screening.

Selecting the best clones, with the proteins interacting most specifically and with highest affinity, can be followed by repeated screenings, thus leading to the most desired target proteins with the highest binding affinity for the query protein. These are suitable for large-scale target protein production.

These aspects and embodiments of the present system are further described in the following examples. However, the present system is not limited by such examples, and variations will be apparent to those skilled in the art without departing from the scope of the present setup.

### Example 1

**Figure 35:**
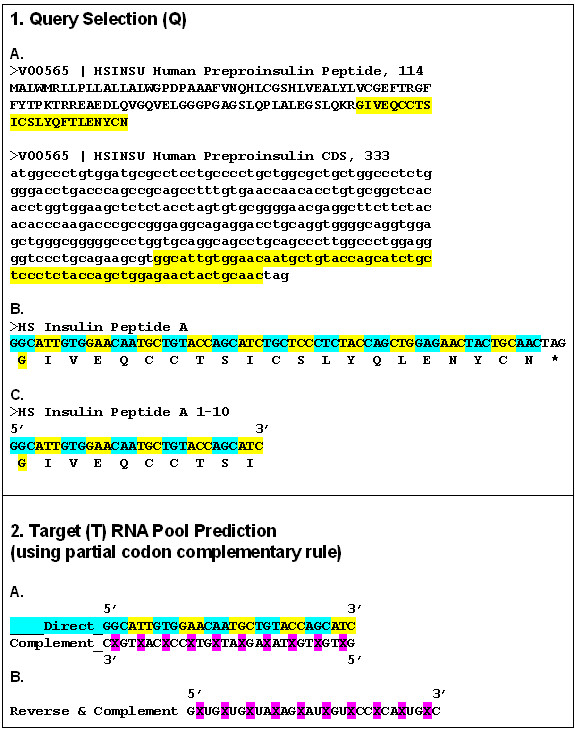
Representative query amino acid sequences and preparation of reverse and complementary sequences, the second nucleotide of each codon in which is replaced with a variable X nucleotide.

Figure 35 shows the use of the present system to obtain a specific high affinity protein with binding affinity for a section of the A-peptide in human insulin.

Starting with the known protein and nucleic acid sequences of the entire Pre-pro-insulin, 1–10 residues of the A peptide and the corresponding nucleic acid sequence are selected. The selected part of the peptide, called the query, is used to screen the target protein expression library. Therefore, this sequence should be available in pure peptide form.

Next, a sequence is created that is complementary to the query nucleotide sequence at the 1st and 3rd codon positions but leaving the 2nd position undefined (X). The complementary sequence is reversed and, in this particular example, the bases T are changed to U. The second (central) codon position remains undefined and this undefined X position can be any one of the possible nucleotides (A, U, G, C). Therefore, this prediction method defines many different target RNA sequences. In the case of a sequence including 30 nucleotide bases, the expected number of possible target sequences will be about 4^10 ^= 10^6^.

**Figure 36:**
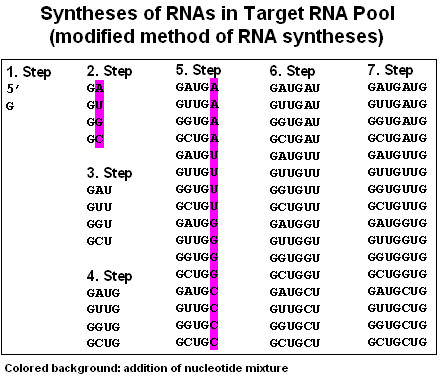
Synthesis pattern for construction of target sequences and the progression of the permutations depending on the number of amino acid residues.

The predicted pool of target RNAs is synthesized by following the usual step-by-step (base by base) protocol, known to those skilled in the art, except the syntheses of the X positions. At the X position, a mixture of nucleotide bases is provided (which contain equal amounts of A and U and G and C). The result of this modified oligo-nucleotide synthesis is a mixture of the desired potential target RNAs as shown in Figure 36. The target RNAs are cloned and transfected via an expression vector into a cell for expression of the encoded protein. An expression library of the expressed target protein is created for screening for query protein/target protein affinity binding. When binding complexes are found to meet the affinity binding levels, the target protein may be cloned for large-scale production.

These steps may be repeated numerous times by modifying the length of the query sequence and/or using another domain area of the query protein that may be of interest.

### Example 2. Example of design and characterization of a specific protein-protein interaction

The BacterioMatch™ two-hybrid system (Stratagene, 11011 N. Torrey Pines Road, La Jolla, CA 92037) was used for quick detection of protein-protein interactions designed by the recent method. It is a simple alternative or complement to yeast two-hybrid systems for detection of such interactions in vivo. Because the two-hybrid assay is performed in bacteria, the results are obtained more easily and quickly than in yeast. The system is based on transcriptional activation of a primary ampicillin-resistant reporter and a secondary β-galactosidase reporter for validation. The BacterioMatch two-hybrid system is based on a methodology developed by Dove, Joung, and Hochschild of Harvard Medical School.

**Figure 37:**
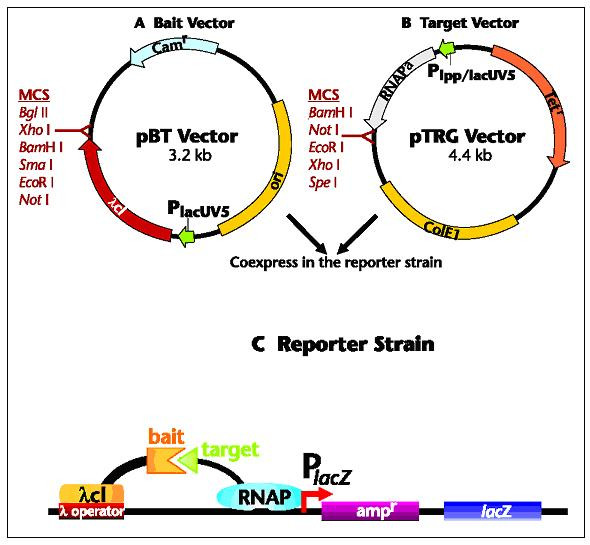
The BacterioMatch™. Two-hybrid system (reproduced from Strategene).

The BacterioMatch two-hybrid system is based on transcriptional activation (Figure 37). A protein of interest – the bait – is fused to the full-length bacteriophage repressor protein (λcI). The corresponding target protein is fused to the amino-terminal domain of the α-subunit of RNA polymerase (RNAPα). The bait is tethered to the x operator sequence upstream of the reporter promoter through the DNA-binding domain of λcI. If the bait and target interact, they recruit and stabilize the binding of RNA polymerase close to the promoter and activate the transcription of the ampicillin-resistant reporter gene in the BacterioMatch two-hybrid reporter strain. The β-galactosidase reporter gene provides an additional mechanism for validating putative protein-protein interactions.

(a) *Bait vector*. The bait vector, pBT, encodes the full-length bacterial phage cI protein under the control of the strong lacUV5 promoter. A protein of interest is fused to the bacterial phage λcI protein by inserting its gene into the multiple cloning site at the 3' end of the λcI gene. The presence of a multiple cloning site makes it convenient to subclone a bait gene that is already present in many yeast two-hybrid bait plasmids.

(b) *Target vector*. The target plasmid, pTRG, is compatible with Stratagene's cDNA library construction kit. The target plasmid directs transcription of the amino-terminal domain of RNA polymerase α-subunit and linker region under the control of tandem promoters lpp and lacUV5. The target gene is fused in-frame to the α-subunit NTD through a multiple cloning site at the 3' end of the α-subunit gene.

(c) *Reporter strain*. The reporter strain is derived from XL1-Blue MRF'. The strain lacks all restriction systems in order to be compatible with current cDNA library construction methods. The lac I^q ^gene, located on the F' episome, represses synthesis of the bait and target until induction. The reporter cassette is also located on the F' episome in the cell. The lacZ gene serves as a secondary reporter to provide a visible phenotype for identifying positive protein-protein interactions.

### Definitions

*Query *(or bait) is one protein sequence with which the target protein, designed and produced by the method, will specifically interact. *Target protein *is one or more protein sequence(s) designed by the method to interact specifically with the query protein sequence. The target is expected to be present in a pool of protein sequences called the *target pool*. The target pool is designed using a *target template*, which is a nucleic acid sequence containing 2/3 defined and 1/3 undefined nucleotides (X). (A target template, which contains 15 undefined nucleic acid residues, will result in 4^15 ^= 10^9 ^different oligonucleotides, which will be translated into the corresponding number of proteins.) The target pool is synthesized using a *target oligo template *(TOT) which has a *constant *(C) and *variable *(V) part. The TOT-C is necessary to synthesize dsDNA of the target pool sequences and it is ~20 nucleotides long. The TOT-V (target template) is about 30–45 nucleotides long, 2/3rd of the nucleotides being unambiguously defined while 1/3rd are not (X). The X residues should be incorporated by adding a mixture of nucleotides (equal amounts of A+T+G+C) to the reaction during oligo synthesis.

The results (numbers of highly, moderately and slightly positive clones) are evaluated by visual inspection. The positive clones are saved for further experiments. If there are no positive clones, it is necessary to validate the orientation and translation frame in the target mRNAs. This is possible by sequencing some target mRNAs. The sequence should show the residue pattern.

Both TARGET TEMPLATE to ESRLERLEQLFLLIF (GAL4 09-23AA) and TARGET TEMPLATE to QLFLLIFPREDLDMI (GAL4 17-31AA) contained numerous positive bacterial clones growing on double selective medium. Sequencing of DNA from the vectors in randomly selected positive clones confirmed that:

- they contained the characteristic TOT pattern, i.e. defined 1st and 3rd codon residues;

- the nucleic acids differed only in the 2nd codon positions; their 1st and 3rd codon positions were identical;

- The restriction endonuclease recognition sequences were present;

- the start and stop codons were present;

- the sequences were inserted into the correct, sense DNA strands;

- the codon frames were correct in relation to the start codon and were read in the correct frames.

Some positive TARGET TEMPLATE to ESRLERLEQLFLLIF (GAL4 09-23AA) clones were further processed to monoclonal colonies and proteins were extracted. Characterization of the binding properties of fluorescently labeled GAL4 peptide to the protein extract indicated the presence of saturable binding sites in the protein extracts from positive clones and the absence of saturable binding sites in the negative clones.

### The experiment

The experiment below is specifically designed for the BacterioMatch (Stratagene) two-hybrid system. This system uses:

- a bait vector (pBT) and the manufacturer's standard as insert, the dimerization domain of 1HBW REGULATORY PROTEIN GAL4;

- a target vector (pTRG) and the manufacturer's standard as insert, and a ~90 aa long mutant form of Gal11.

In the experiment below the target oligo pool is used instead of Gall1 in the pTRG vector.

The query in this experiment is the dimerization domain of 1HBW REGULATORY PROTEIN GAL4 inserted into pBT (as provided and described by Stratagene). The target oligo templates (TOT-V) were designed to interact specifically with K01486_SCGAL4_DIMDOM-171/9-23 and K01486_SCGAL4_DIMDOM-171/17/31 sequences.

**Figure 38:**
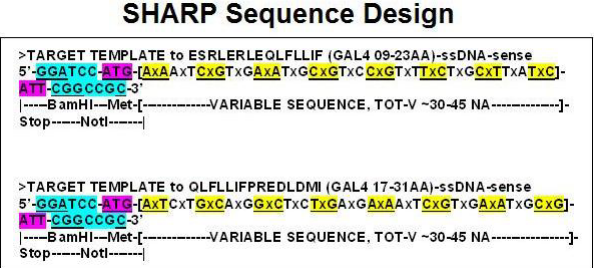
Sequences designed by this method were expected to produce proteins (when transcribed and translated) with the potential to interact specifically with the indicated domains of the Gal4 protein. The 1st and 3rd codon letters in these target templates are complementary to the 3rd and 1st codon letters in the Gal4 coding sequences (reverse reading direction) while the 2nd codon letter is undefined (A or T or G or C).

The sequences below are sense, ssDNA sequences, which means that the TOT-V in this sequence is the same as the sequence in the expected mRNAs (except for T/U conversion). The TOT-C is not indicated here; BPD can decide which TOT-C to use for this purpose (Figure 38).

**Figure 39:**
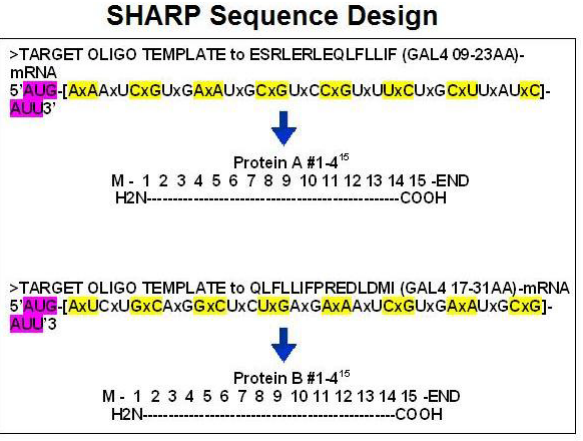
The transcription of TOT dsDNA will result in TOT mRNA. A 45 nucleotide long TOT will be translated into 4^15 ^different oligopeptides, each 15 amino acids long. Some of these oligopeptides are expected to interact specifically with the respective GAL4 targets.

The experiment consists of the following steps:

(1) Sequence the Gal 4 DNA (provided by Stratagene) to make sure that the query sequence is as expected.

(2) Synthesize the target pool using the target oligo templates. This is a single run routine oligo synthesis. Residue X is equal amounts of A+T+G+C.

(3) Make dsDNAs. This is a single run PCR.

(4) Make restriction enzyme cuts on the target oligo pool sequences. This is a single run RE reaction.

(5) Insert the oligo pool sequences into the pTRG vector. ~10^9 ^different vectors are expected. Make sure that the orientation of the target oligos is correct and the transcription will result in the following mRNA. The target oligo pool insertion is a single run ligase reaction (Figure 39). Transcription of TOT dsDNA will result in TOT mRNA. A 45 nucleotide long TOT will be translated into 4^15 ^different oligopeptides, each 15 amino acids long. Some of these oligopeptides are expected to interact specifically with the respective GAL4 targets.

(6) Insert the vectors into bacteria.

(7) Perform the BacterioMatch two-hybrid assay accordingly to the Stratagene manual.

The *K*_d _of the binding sites varied between 1 and 100 nM, indicating the presence of a limited number of high affinity binding sites. Unlabelled GAL4 inhibited the binding of labeled GAL4 to the proteins from positive clones while other randomly chosen proteins (insulin, growth hormone, prolactin) were ineffective competitors even in much higher concentrations.

This experiment indicates that it is possible to design specifically interacting oligo-peptides (target) to any oligo-peptide (query) and detect the interaction in a bacterial two-hybrid system (like BacterioMatch™. The method is quick; it takes only a few days to obtain interacting monoclonal proteins. The designed protein-protein interaction is highly specific and has high affinity (*K*_d _~ 1–100 nM).

Further details can be found in the following references [58,59,98].

### SHARHs compared to other affinity reagent designs

Really *ab initio *design of affinity peptides (antibodies, receptors) is not yet possible, because the rules of specific protein-protein interactions are not well understood. Therefore random protein library constructions and affinity screenings are used as alternative methods to the classical immunization techniques.

Affibodies (Affibody of Bromma in Sweden) were among the first non-immunoglobulin-based affinity reagents. These small molecules are based on a bacterial receptor (Staphylococcus aureus protein A), and use combinatorial protein engineering to introduce random mutations in the affinity region. [99].

Another non-immunoglobulin-based affinity reagent that is becoming more widely used is the aptamer. Made of DNA, RNA or modified nucleic acids and typically 15–40 bases in length, aptamers have a stable tertiary structure that permits protein binding through van der Waals forces, hydrogen bonding and electrostatic interactions. Early studies showed that aptamers can be highly specific for target proteins, with the ability to distinguish between related members of a protein family [100].

No one of the above mentioned methods is able to satisfy the emerging need to produce affinity reagents at a truly high-throughput scale. A typical random peptide library has about a billion phage clones – enough to represent most of the 4^18 ^possible 6-mers, but far too small to represent the 4^45 ^possible 15-mers. Therefore the definition of 1^st ^and 3^rd ^codons accordingly to the Proteomic Code is a big help to overcome these size limitation. The proteomic Code makes it possible to design and produce 15-mers in typical libraries (4^15 ^~ 10^9^).

What about if the concept of the Proteomic Code is wrong? The method to design and produce SHARP peptides in partially random libraries remains still plausible. The characteristics of amino acids are largely determined by the base on the second codon position and the bases on the second positions are undefined or randomly selected when using the SHARP method.

## Conclusion

### The promises of the second generation Proteomic Code

#### Industrial applications

The second generation Proteomic Code and the method for developing SHARP have potential advantages that are not obvious in the recent antibody-developing methods:

- provide quick access to interacting peptides;

- provide direct and permanent access to monoclonal sources for large-scale production;

- SHARP is small (MW <2000 Da) compared to antibodies (155 kDa) or affibodies (which gives therapeutic and manufacturing advantage), no need for humanization;

- might be the key to mass production of interactive oligopeptides (similar to the on-demand synthesis of nucleotide oligos;

- a self-learning method; every single successful SHARP can contribute to a more and more exact amino acid interaction table.

#### Scientific potential

The present system is a unique in silico method for identifying the binding proteins that interact most effectively with reactive epitopes on a respective protein antigen. The system has widespread applications and is beneficial to biotechnology. It is useful, for example, in developing drugs for treating viral diseases such as AIDS and influenza, as well as diseases such as Alzheimer's disease and bovine spongiform encephalopathy. In addition to medical research and drug development, this system has applications related to environmental health and public safety, including for example the detection of bacteria, viruses, toxins, etc. in air, water and food supplies.

By way of further specific examples, the present system has applications in the following areas:

1. improving health care, by providing a new and easily implemented approach to the development of diagnostic kits and therapeutic drugs;

2. improving the environment, by providing new and economic approaches for detecting environmental pathogens;

3. improving working conditions by providing economic and effective ways of detecting environmental pathogens; and

4. improving homeland security by providing rapid detection of known as well as new pathogens in air, water, food, etc.

### The vision of a proteome-sensor chip

Detection and measurement of proteins is a fundamental procedure in life sciences. Many diagnostic procedures are already based on this technique and many more will follow:

- detection of hormones and enzymes for diagnosis of organ failure;

- detection of pathogen-derived antigens or antibody responses for diagnosis of infections;

- detection of allergens for allergy diagnoses;

- tumor markers to detect and evaluate neoplasias.

Considering that the number of hormones, enzymes, pathogens, and markers is very large, it is easy to recognize that the demand for specifically interacting diagnostic proteins is large. However, there are limitations to satisfaction of the demand. It takes several weeks to develop one antibody by traditional methods. Therefore it is not cheap. The traditional one protein-one kit method is simply no longer feasible. Some kind of integration is necessary. Development points to protein chips that permit the simultaneous, parallel detection of hundreds or thousands of protein signals and the computerized integrated evaluation of the results. Chip-based protein detection requires a large number of easy-to-produce, cheap, interacting proteins.

The Proteomic Code-based method described here could contribute significantly to the industrial production of such interacting peptides.

**Figure 40:**
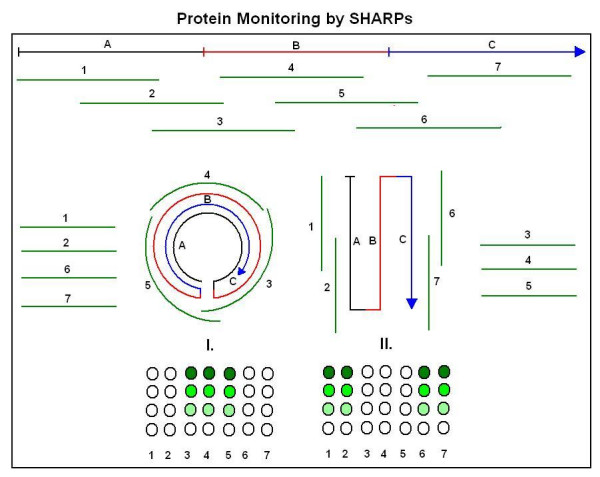
Protein monitoring by SHARPs. Specific and high affinity reacting proteins (SHARPs, 1–7) are designed and produced against a protein. The protein has 3 domains (A, B, C) and two main conformations (I, II). Domain B is exposed on the surface in conformation I and that form is detected by SHARPs 3–5. Domain B is sandwiched between domains A and C in configuration II and therefore this form is detected by SHARPs 1–2 and 6–7. A concentration and configuration dependent response is detected by a slot corresponding to the protein by the SHARPs CHIP (dots).

The SHARP chip-based technology opens the way to the real possibility of monitoring the proteome, i.e. obtaining detailed information on the qualitative and quantitative state of a large number of different proteins simultaneously, even including some information about the splicing and configuration changes in individual proteins (Figure 40).

The human genome contains 3 × 10^9 ^base pairs. About 2% of this is located in about 30,000 genes that are expressed, spliced into ~100,000 different proteins containing ~40 million amino acids. The 2× coverage of this size proteome with 15-residue complementary sequences requires 5 million different oligopeptides. (This is probably the upper estimate, because the proteome contains many similar or identical domains.) Proteome monitoring with proteomic chips is a huge challenge, which can only be met if SHARP proteins become accessible in much larger number and for a much lower price than is possible today.

### The vision of a new physiology

The receptor – ligand and antigen – antibody type or interaction has a fundamental role in the physiology of humans and animals. Obviously, peptides that can interact specifically with regulatory pathways have significant potential for manipulating these pathways. Most drugs are effective because of their interaction with regulatory pathways (e.g., GPCR or 7TM receptor systems). Therefore, easy and inexpensive access to designed interacting proteins will have an impact on further development of physiological and pharmacological research and drug discovery. Some kind of industrial-scale, standardized, semi-automated physiological research is also desirable. The traditional one-by-one approach is too slow and too expensive for the complexity of life.

### The vision of new protein-based therapeutic approaches

Proteins/peptides are underutilized in medical therapy. Only insulin is used to treat a common disease (Type I diabetes); a few other proteins are used to treat relatively rare diseases such as growth hormone (GH) deficiency and hemophilia. Veterinary use of GH (lean meat production) or misuse in sports and cosmetology far exceeds its medical indication. Protein therapy is expensive and requires daily injections, which is not attractive to most patients. Most pharmaceutical companies have accumulated large bodies of knowledge (and patent bases) regarding traditional, simple molecular drug design and treatments, while their knowledge in proteomics is still undergoing development. Simple molecule-based drugs do not provide much specificity. Protein-based, highly specific treatments are not too far off in the future and easy access to biologically active proteinaceous substances will facilitate physiological evaluation and medical application of these more complex peptide molecules.

SHARPs, complementary or not, are obvious candidates for receptor agonist or antagonist functions. However, there is an even more exciting and less expected therapeutic application of complementary peptides. Some experiments suggest that immunization with complementary peptides to receptors induces production of ligand-like antibodies, with ligand-like biological effects. Just imagine the possibility of inducing insulin-like antibodies by immunization with designed complementary peptides to insulin-receptors and obtaining insulin-like effects (regulation will of course be a problem to solve); or treating GH deficiency by raising GH-like antibodies using GH-receptor complementary peptides for immunization. This seems like science fiction today, but Blalocks' experiments, for example, are already pointing in that direction [27].

Is protein therapy an alternative to gene therapy? Yes. Gene therapy is technically still difficult to perform and the effect is irreversible. The effects of protein therapy are short-lived and reversible (if immunization does not occur).

## Software

SeqForm [78], SeqPlot [79], Dotlet, Mfold [64], SeqX [57].

## Patents

Blalock [101], Omichinski [102], Biro [103].

## Abbreviations

amino acid complementarity: the physico-chemical complementarity (size, charge, hydropathy) of amino acid pairs as well as their origin from complementary codons

complementary amino acids: amino acid pairs, coded by complementary codons

NA: nucleic acid

P: protein

PC: Proteomic Code, the comprehensive rules describing the origin and nature of amino acid complementarity

SHARP: Specific High Affinity Reacting Peptides

W-C base pairs: Watson-Crick's complementary base pairs.

## Competing interests

The author of this article has pending US and PCT patent applications for the system and the method of obtaining oligo-peptides with specific high affinity to query proteins.

## Authors' contributions

JC Biro is the sole author of this review

## Supplementary Material

Additional file 1 Experiments related to Proteomic Code. Collection of experiments and references related to Proteomic Code. Click here for file

Additional file 2 List of Amino Acid Pairs, Proteomic Codes & Physicochemical Properties. List of Amino Acid Pairs, Proteomic Codes & Physicochemical Properties. Click here for file
