# Supplementary material for: The Proteomic Code: a molecular recognition code for proteins
Source: Theor Biol Med Model. 2007 Nov 13;4:45. doi: 10.1186/1742-4682-4-45 (PMC2206014; doi:10.1186/1742-4682-4-45)
Supplement: Additional file 1 — Experiments related to Proteomic Code. Collection of experiments and references related to Proteomic Code. [file 1742-4682-4-45-S1.doc]

**Additional File 1**


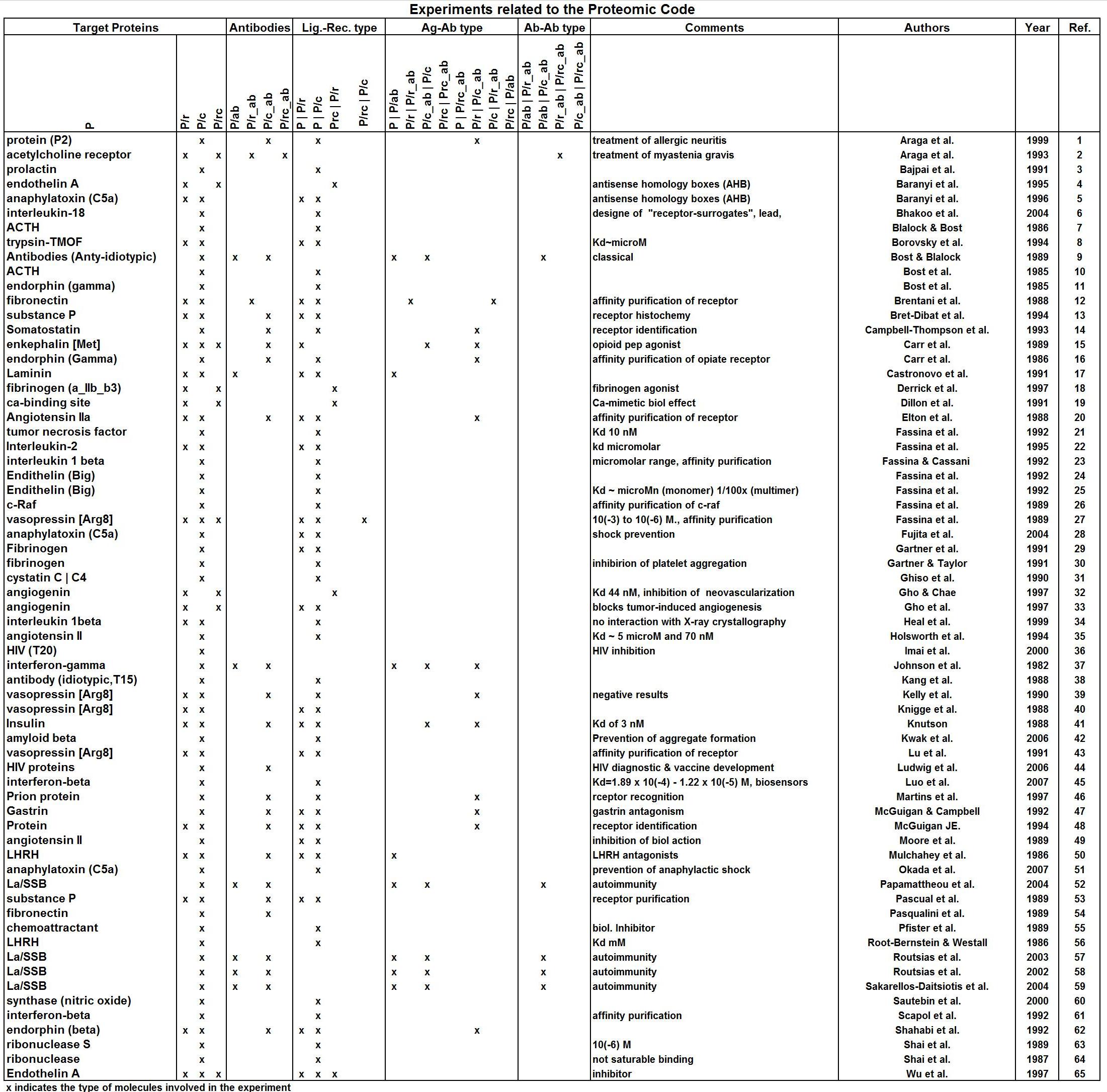


**References to Table**

1. Araga S, Kishimoto M, Doi S, Nakashima K: **A complementary peptide vaccine that induces T cell anergy and prevents experimental allergic neuritis in Lewis rats.** *J Immunol* 1999, **163:**476-482.

2. Araga S, LeBoeuf RD, Blalock JE: **Prevention of experimental autoimmune myasthenia gravis by manipulation of the immune network with a complementary peptide for the acetylcholine receptor**. *Proc Natl Acad Sci USA* 1993, **90:**8747-8751.

3. Bajpai A, Hooper KP, Ebner KE: **Interactions of antisense peptides with ovine prolactin.** *Biochem Biophys Res Commun* 1991, **180:**1312-1317.

4. Baranyi L, Campbell W, Ohshima K, Fujimoto S, Boros M, Okada H: **The antisense homology box: a new motif within proteins that encodes biologically active peptides.** *Nat Med* 1995, **1:**894-901.

5. Baranyi L, Campbell W, Okada H: **Antisense homology boxes in C5a receptor and C5a anaphylatoxin: a new method for identification of potentially active peptides.** *J Immunol* 1996, **157:**4591-4601.

6. [Bhakoo A](http://www.ncbi.nlm.nih.gov/sites/entrez?Db=pubmed&Cmd=Search&Term="Bhakoo A"%5BAuthor%5D&itool=EntrezSystem2.PEntrez.Pubmed.Pubmed_ResultsPanel.Pubmed_RVAbstract), [Raynes JG](http://www.ncbi.nlm.nih.gov/sites/entrez?Db=pubmed&Cmd=Search&Term="Raynes JG"%5BAuthor%5D&itool=EntrezSystem2.PEntrez.Pubmed.Pubmed_ResultsPanel.Pubmed_RVAbstract), [Heal JR](http://www.ncbi.nlm.nih.gov/sites/entrez?Db=pubmed&Cmd=Search&Term="Heal JR"%5BAuthor%5D&itool=EntrezSystem2.PEntrez.Pubmed.Pubmed_ResultsPanel.Pubmed_RVAbstract), [Keller M](http://www.ncbi.nlm.nih.gov/sites/entrez?Db=pubmed&Cmd=Search&Term="Keller M"%5BAuthor%5D&itool=EntrezSystem2.PEntrez.Pubmed.Pubmed_ResultsPanel.Pubmed_RVAbstract), [Miller AD](http://www.ncbi.nlm.nih.gov/sites/entrez?Db=pubmed&Cmd=Search&Term="Miller AD"%5BAuthor%5D&itool=EntrezSystem2.PEntrez.Pubmed.Pubmed_ResultsPanel.Pubmed_RVAbstract).
De-novo design of complementary (antisense) peptide mini-receptor inhibitor of interleukin 18 (IL-18).
[Mol Immunol.](javascript:AL_get(this, 'jour', 'Mol Immunol.');) 2004 Nov; 41(12):1217-24.

7. Blalock JE, Bost KL: **Binding of peptides that are specified by complementary RNAs.** *Biochem J* 1986, **234:**679-683.

8. Borovsky D, Powell CA, Nayar JK, Blalock JE, Hayes TK: **Characterization and localization of mosquito-gut receptors for trypsin modulating oostatic factor using a complementary peptide and immunocytochemistry.** *FASEB J* 1994, **8:**350-355.

9. Bost KL, Blalock JE: **Production of anti-idiotypic antibodies by immunization with a pair of complementary peptides.** *J Mol Recognit* 1989, **1:**179-183.

10. Bost KL, Smith EM, Blalock JE: **Similarity between the corticotrophin (ACTH) receptor and a peptide encoded by an RNA that is complementary to ACTH mRNA.** *Proc Natl Acad Sci USA* 1985, **82:**1372-1375.

11. Bost KL, Smith EM, Blalock JE: **Similarity between the corticotrophin (ACTH) receptor and a peptide encoded by an RNA that is complementary to ACTH mRNA.** *Proc Natl Acad Sci USA* 1985, **82:**1372-1375.

12. Brentani RR, Ribeiro SF, Potocnjak P, Pasqualini R, Lopes JD, Nakaie CR: **Characterization of the cellular receptor for fibronectin through a hydropathic complementarity approach.** *Proc Natl Acad Sci USA* 1988, **85:**364-367.

13. Bret-Dibat JL, Zouaoui D, Déry O, Zerari F, Grassi J, Maillet S, Conrath M, Couraud JY: **Antipeptide polyclonal antibodies that recognize a substance P-binding site in mammalian tissues: a biochemical and immunocytochemical study.** *J Neurochem* 1994, **63:**333-343.

14. Campbell-Thompson M, McGuigan JE: **Canine parietal cell binding by antibodies to the complementary peptide of somatostatin**. *Am J Med Sci* 1993, **305:**365-373.

15. Carr DJ, Blalock JE, Bost KL: **Monoclonal antibody against a peptide specified by [Met]-enkephalin complementary RNA recognizes the delta-class opioid receptor.** *Immunol Lett* 1989, **20:**181-186.

16. Carr DJ, Bost KL, Blalock JE: **An antibody to a peptide specified by an RNA that is complementary to gamma-endorphin mRNA recognizes an opiate receptor.** *J Neuroimmunol* 1986, **12:**329-337.

17. Castronovo V, Taraboletti G, Sobel ME: **Laminin receptor complementary DNA-deduced synthetic peptide inhibits cancer cell attachment to endothelium.** *Cancer Res* 1991, **51:**5672-5678.

18. Derrick JM, Taylor DB, Loudon RG, Gartner TK: **The peptide LSARLAF causes platelet secretion and aggregation by directly activating the integrin alphaIIbbeta3.** *Biochem J* 1997, **325(Pt 2):**309-313.

19. Dillon J, Woods WT, Guarcello V, LeBoeuf RD, Blalock JE: **A peptide mimetic of calcium.** *Proc Natl Acad Sci USA* 1991, **88:**9726-9729.

20. Elton TS, Oparil S, Blalock JE: **The use of complementary peptides in the purification of an angiotensin II binding protein.** *J Hypertens* 1988, **6(Suppl):**S404-407.

21. Fassina G, Cassani G, Corti A: **Binding of human tumor necrosis factor alpha to multimeric complementary peptides.** *Arch Biochem Biophys* 1992, **296:**137-143.

22. Fassina G, Cassani G, Gnocchi P, Fornasiero MC, Isetta AM: **Inhibition of interleukin-2/p55 receptor subunit interaction by complementary peptides.** *Arch Biochem Biophys* 1995, **318:**37-45.

23. Fassina G, Cassani G, Corti A: **Binding of human tumor necrosis factor alpha to multimeric complementary peptides.** *Arch Biochem Biophys* 1992, **296:**137-143.

24. Fassina G, Consonni R, Zetta L, Cassani G. **Design of hydropathically complementary peptides for Big Endothelin affinity purification**. *Int J Peptide Protein Res* 1992, **39:**540-548.

25. Fassina G, Corti A, Cassani G: **Affinity enhancement of complementary peptide recognition.** *Int J Peptide Protein Res* 1992, **39:**549-556.

26. Fassina G, Roller PP, Olson AD, Thorgeirsson SS, Omichinski JG: **Recognition properties of peptides hydropathically complementary to residues 356-375 of the c-raf protein.** *J Biol Chem* 1989, **264:**11252-11257.

27. Fassina G, Zamai M, Brigham-Burke M, Chaiken IM: **Recognition properties of antisense peptides to Arg8-vasopressin/bovine neurophysin II biosynthetic precursor sequences.** *Biochemistry* 1989, **28:**8811-8818.

28. Fujita E, Farkas I, Campbell W, Baranyi L, Okada H, Okada N: **Inactivation of C5a anaphylatoxin by a peptide that is complementary to a region of C5a**. *J Immunol* 2004, **172:**6382-6387.

29. Gartner TK, Loudon R, Taylor DB: **The peptides APLHK, EHIPA and GAPL are hydropathically equivalent peptide mimics of a fibrinogen binding domain of glycoprotein IIb/IIIa.** *Biochem Biophys Res Commun* 1991, **180:**1446-1452.

30. Gartner TK, Taylor DB: **The peptide Glu-His-Ile-Pro-Ala binds fibrinogen and inhibits platelet aggregation and adhesion to fibrinogen and vitronectin.** *Proc Soc Exp Biol Med* 1991, **198:**649-655.

31. Ghiso J, Saball E, Leoni J, Rostagno A, Frangione B: **Binding of cystatin C to C4: the importance of sense-antisense peptides in their interaction.** *Proc Natl Acad Sci USA* 1990, **87:**1288-1291.

32. Gho YS, Chae CB: **Anti-angiogenin activity of the peptides complementary to the receptor-binding site of angiogenin.** *J Biol Chem* 1997, **272:**24294-24299.

33. Gho YS, Lee JE, Oh KS, Bae DG, Chae CB: **Development of antiangiogenin peptide using a phage-displayed peptide library.** *Cancer Res* 1997, **57:**3733-3340.

34. Heal JR, Bino S, Ray KP, Christie G, Miller AD, Raynes JG: **A search within the IL-1 type I receptor reveals a peptide with hydropathic complementarity to the IL-1beta trigger loop which binds to IL-1 and inhibits in vitro responses.** *Mol Immunol* 1999, **36:**1141-1148.

35. Holsworth DD, Kiely JS, Root-Bernstein RS, Overhiser RW: **Antisense-designed peptides: a comparative study focusing on possible complements to angiotensin II.** *Peptide Res* 1994, **7:**185-193.

36. Imai M, Okada N, Okada H: **Inhibition of HIV-1 infection by an intramolecular antisense peptide to T20 in gp160.** *Microbiol Immunol* 2000, **44:**205-12.

37. Johnson HM, Langford MP, Lakhchaura B, Chan TS, Stanton GJ: **Neutralization of native human gamma interferon (HuIFN gamma) by antibodies to a synthetic peptide encoded by the 5' end of HuIFN gamma cDNA.** *J Immunol* 1982, **129:**2357-2359.

38. Kang CY, Brunck TK, Kieber-Emmons T, Blalock JE, Kohler H: **Inhibition of self-binding antibodies (autobodies) by a VH-derived peptide.** *Science* 1988, **240:**1034-1036.

39. Kelly JM, Trinder D, Phillips PA, Casley DJ, Kemp B, Mooser V, Johnston CI: **Vasopressin antisense peptide interactions with the V1 receptor.** *Peptides* 1990, **11:**857-862.

40. Knigge KM, Piekut DT, Berlove D: **Immunocytochemistry of a vasopressin (AVP) receptor with anti-idiotype antibody: inhibition of staining with a peptide (PVA) encoded by an RNA that is complementary to AVP mRNA.** *Neurosci Lett* 1988, **86:**269-271.

41. Knutson VP: **Insulin-binding peptide. Design and characterization.** *J Biol Chem* 1988, **263:**14146-14151.

42. Kwak JW, Kim HK, Chae CB: **Potential lead for an Alzheimer drug: a peptide that blocks intermolecular interaction and amyloid beta protein-induced cytotoxicity.** *J Med Chem* 2006, **49:**4813-4817.

43. Lu FX, Aiyar N, Chaiken I: **Affinity capture of [Arg8]vasopressin-receptor complex using immobilized antisense peptide.** *Proc Natl Acad Sci USA* 1991, **88:**3642-3646.

44. Ludwig LB, Ambrus JL, Krawczyk KA, Sharma S, Brooks S, Hsiao CB, Schwartz SA: **Human Immunodeficiency Virus-Type 1 LTR DNA contains an intrinsic gene producing antisense RNA and protein products.** *Retrovirology* 2006, **3:**80.

45. Luo J, Zhang Q, Huang Y, Liu G, Zhao R: **Quartz crystal microbalance biosensor for recombinant human interferon-beta detection based on antisense peptide approach.** *Anal Chim Acta* 2007, **590:**91-97.

46. Martins VR, Graner E, Garcia-Abreu J, de Souza SJ, Mercadante AF, Veiga SS, Zanata SM, Neto VM, Brentani RR: **Complementary hydropathy identifies a cellular prion protein receptor.** *Nat Med* 1997, **3:**1376-1382.

47. McGuigan JE, Campbell-Thompson M: **Complementary peptide to the carboxyl-terminal tetrapeptide of gastrin.** *Gastroenterology* 1992, **103:**749-758.

48. McGuigan JE: **Antibodies to complementary peptides as probes for receptors.** *Immunomethods* 1994, **5:**158-166.

49. Moore GJ, Ganter RC, Franklin KJ: **Angiotensin 'antipeptides': (-)messenger RNA complementary to human angiotensin II (+)messenger RNA encodes an angiotensin receptor antagonist.** *Biochem Biophys Res Commun* 1989 **160:**1387-1391.

50. Mulchahey JJ, Neill JD, Dion LD, Bost KL, Blalock JE: **Antibodies to the binding site of the receptor for luteinizing hormone-releasing hormone (LHRH): generation with a synthetic decapeptide encoded by an RNA complementary to LHRH mRNA.** *Proc Natl Acad Sci USA* 1986, **83:**9714-718.

51. Okada N, Asai S, Hotta A, Miura N, Ohno N, Farkas I, Hau L, Okada H: **Increased inhibitory capacity of an anti-C5a complementary peptide following acetylation of N-terminal alanine.** *Microbiol Immunol* 2007, **1:**439-443.

52. Papamattheou MG, Routsias JG, Karagouni EE, Sakarellos C, Sakarellos-Daitsiotis M, Moutsopoulos HM, Tzioufas AG, Dotsika EN: **T cell help is required to induce idiotypic-anti-idiotypic autoantibody network after immunization with complementary epitope 289-308aa of La/SSB autoantigen in non-autoimmune mice.** *Clin Exp Immunol* 2004, **135:**416-426.

53. Pascual DW, Blalock JE, Bost KL: **Antipeptide antibodies that recognize a lymphocyte substance P receptor.** *J Immunol* 1989, **143:**3697-3702.

54. Pasqualini R, Chamone DF, Brentani RR: **Determination of the putative binding site for fibronectin on platelet glycoprotein IIb-IIIa complex through a hydropathic complementarity approach.** *J Biol Chem* 1989, **264:**14566-14570.

55. Pfister RR, Haddox JL, Blalock JE, Sommers CI, Coplan L, Villain M: *Synthetic complementary peptides inhibit a neutrophil chemoattractant found in the alkali-injured cornea.* *Cornea* 2000, **19:**384-389.

56. Root-Bernstein RS, Westall FC: **Bovine pineal antireproductive tripeptide binds to luteinizing hormone-releasing hormone: a model for peptide modulation by sequence specific peptide interactions?** *Brain Res Bull* 1986, **17:**519-528.

57. Routsias JG, Dotsika E, Touloupi E, Papamattheou M, Sakarellos C, Sakarellos-Daitsiotis M, Moutsopoulos HM, Tzioufas AG: **Idiotype-anti-idiotype circuit in non-autoimmune mice after immunization with the epitope and complementary epitope 289-308aa of La/SSB: implications for the maintenance and perpetuation of the anti-La/SSB response.** *J Autoimmun* 2003, **21:**17-26.

58. Routsias JG, Touloupi E, Dotsika E, Moulia A, Tsikaris V, Sakarellos C, Sakarellos-Daitsiotis M, Moutsopoulos HM, Tzioufas AG: **Unmasking the anti-La/SSB response in sera from patients with Sjogren's syndrome by specific blocking of anti-idiotypic antibodies to La/SSB antigenic determinants.** *Mol Med* 2002, **8:**293-305.

59. Sakarellos-Daitsiotis M, Cung MT, Sakarellos C, El Hilali Z, Kosmopoulou A, Voitharou C: **Complementary peptide epitopes and anti-idiotypic antibodies in autoimmunity.** *Protein Peptide Lett* 2004, **11:**367-375.

60. Sautebin L, Rombolà L, Di Rosa M, Caliendo G, Perissutti E, Grieco P, Severino B, Santagada V: **Synthesis and structure-activity of antisense peptides corresponding to the region for CaM-binding domain of the inducible nitric oxide synthase.** *Eur J Med Chem* 2000, **35:**727-732.

61. Scapol L, Rappuoli P, Viscomi GC: **Purification of recombinant human interferon-beta by immobilized antisense peptides.** *J Chromatogr* 1992, **600:**235-242.

62. Shahabi NA, Bost KL, Madhok TC, Sharp BM: **Characterization of antisera to the naloxone-insensitive receptor for beta-endorphin on U937 cells generated by using the complementary peptide strategy.** *J Pharmacol Exp Ther* 1992, **263:**876-883.

63. Shai Y, Brunck TK, Chaiken IM: **Antisense peptide recognition of sense peptides: sequence simplification and evaluation of forces underlying the interaction.** *Biochemistry* 1989, **28:**8804-8811.

64. Shai Y, Flashner M, Chaiken IM: **Anti-sense peptide recognition of sense peptides: direct quantitative characterization with the ribonuclease S-peptide system using analytical high-performance affinity chromatography.** *Biochemistry* 1987, **26:**669-675.

65. Wu X, Richards NT, Johns EJ, Kohsaka T, Nakamura A, Okada H: **Influence of ETR-p1/f1 antisense peptide on endothelin-induced constriction in rat renal arcuate arteries.** *Br J Pharmacol* 1997, **122:**316-320.
