# Supplementary figures and images for: The Proteomic Code: a molecular recognition code for proteins
Source: Theor Biol Med Model. 2007 Nov 13;4:45. doi: 10.1186/1742-4682-4-45 (PMC2206014; doi:10.1186/1742-4682-4-45)

**Additional File 2**

**List of Amino Acid Pairs, Proteomic Codes & Physicochemical Properties**


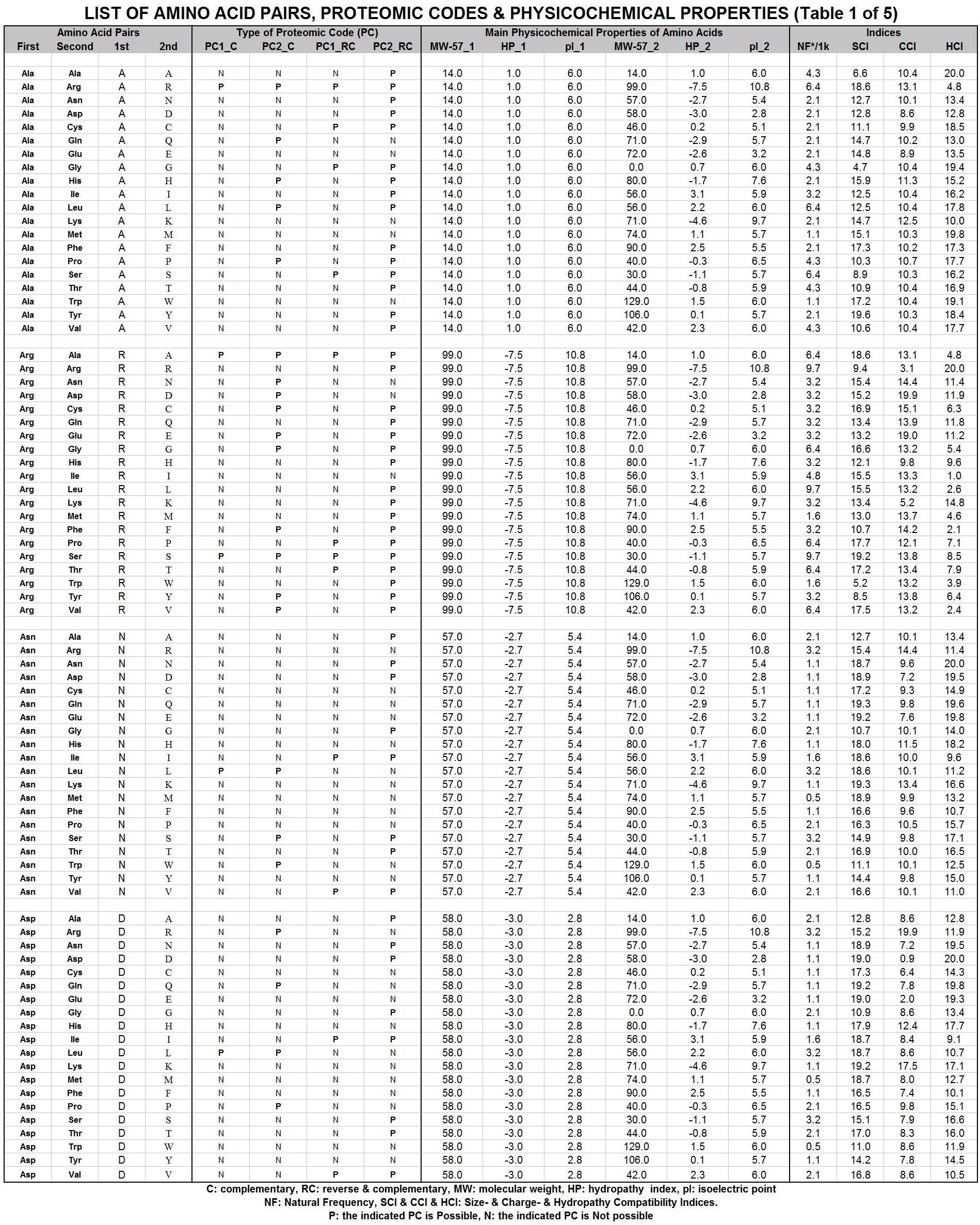


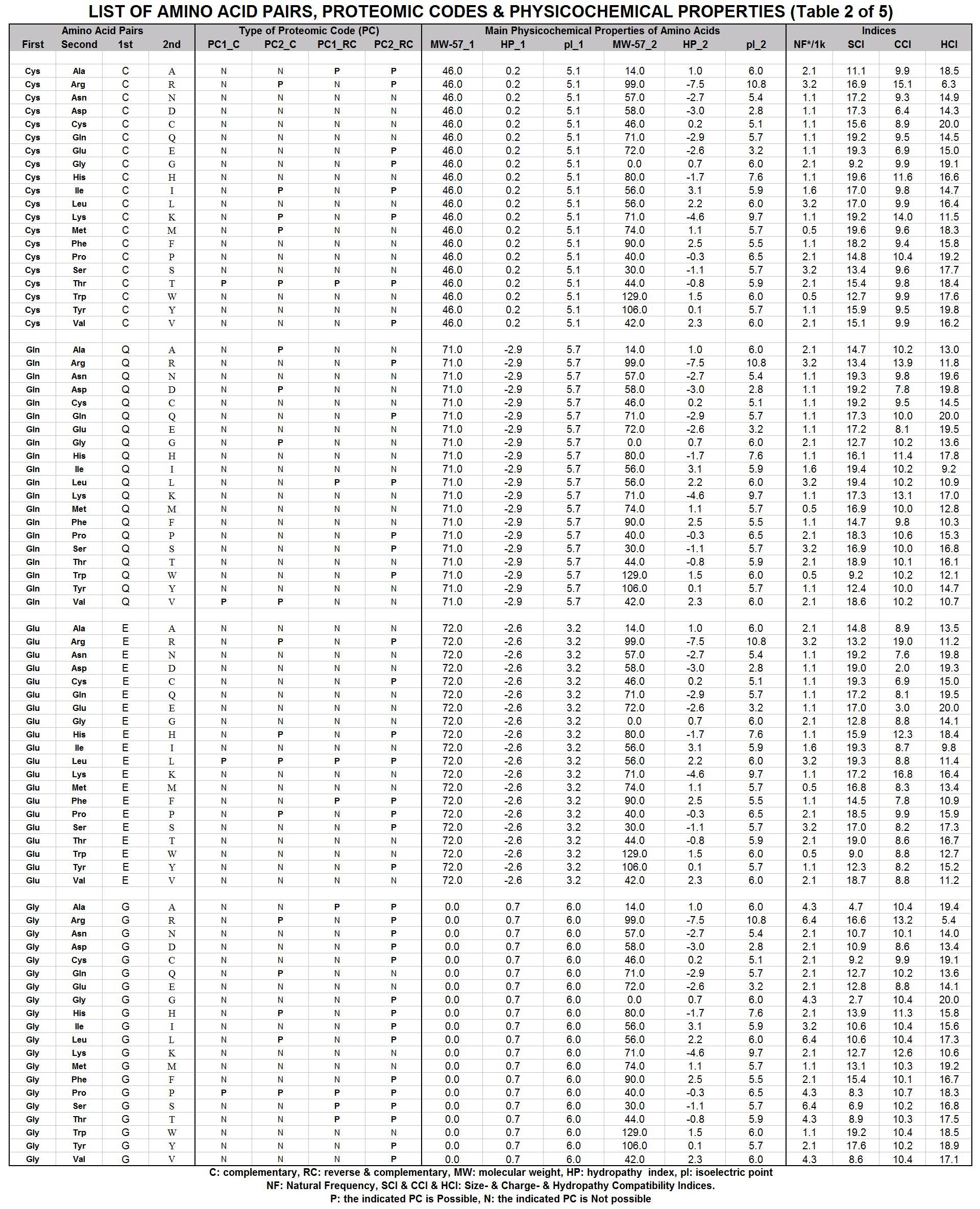


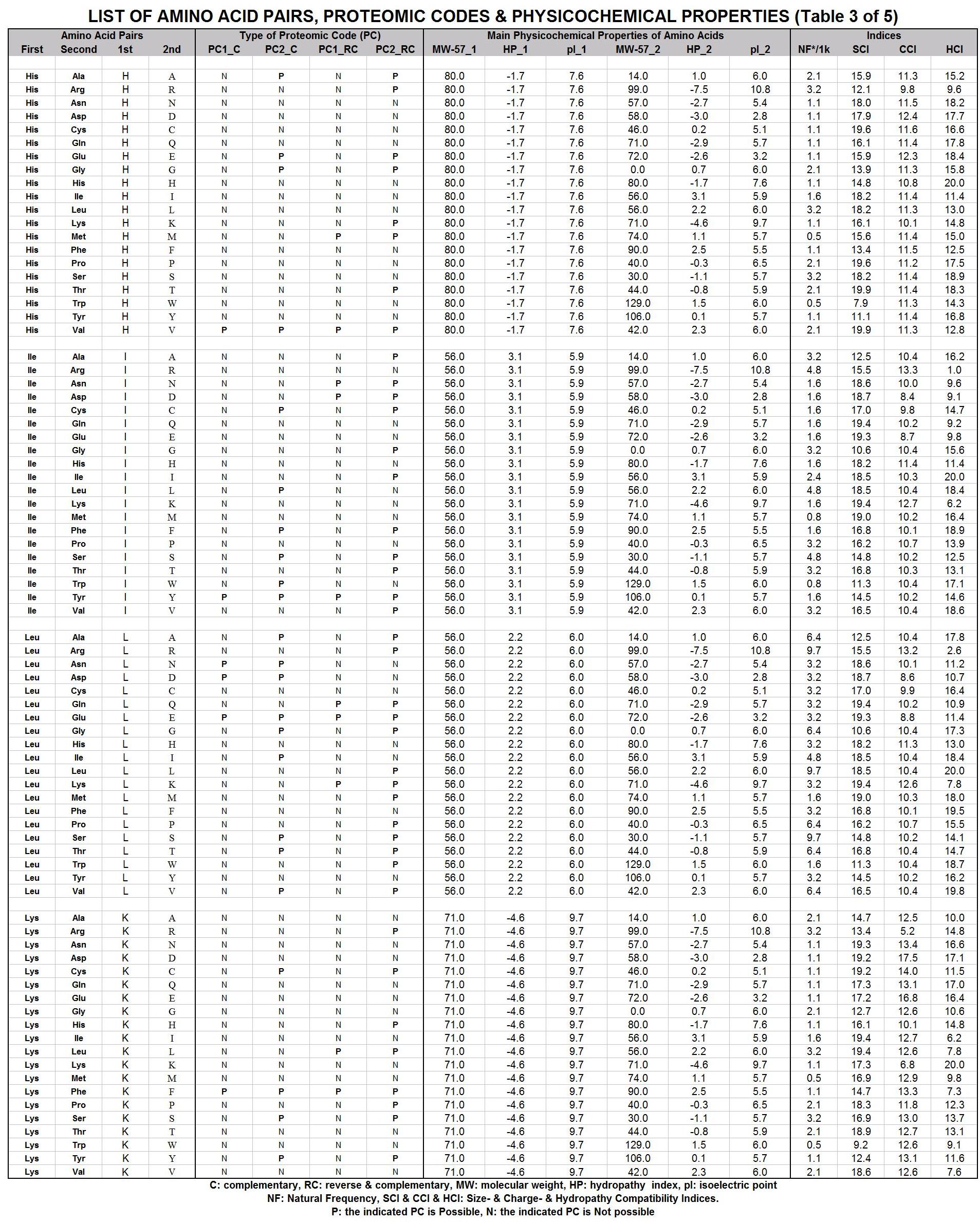


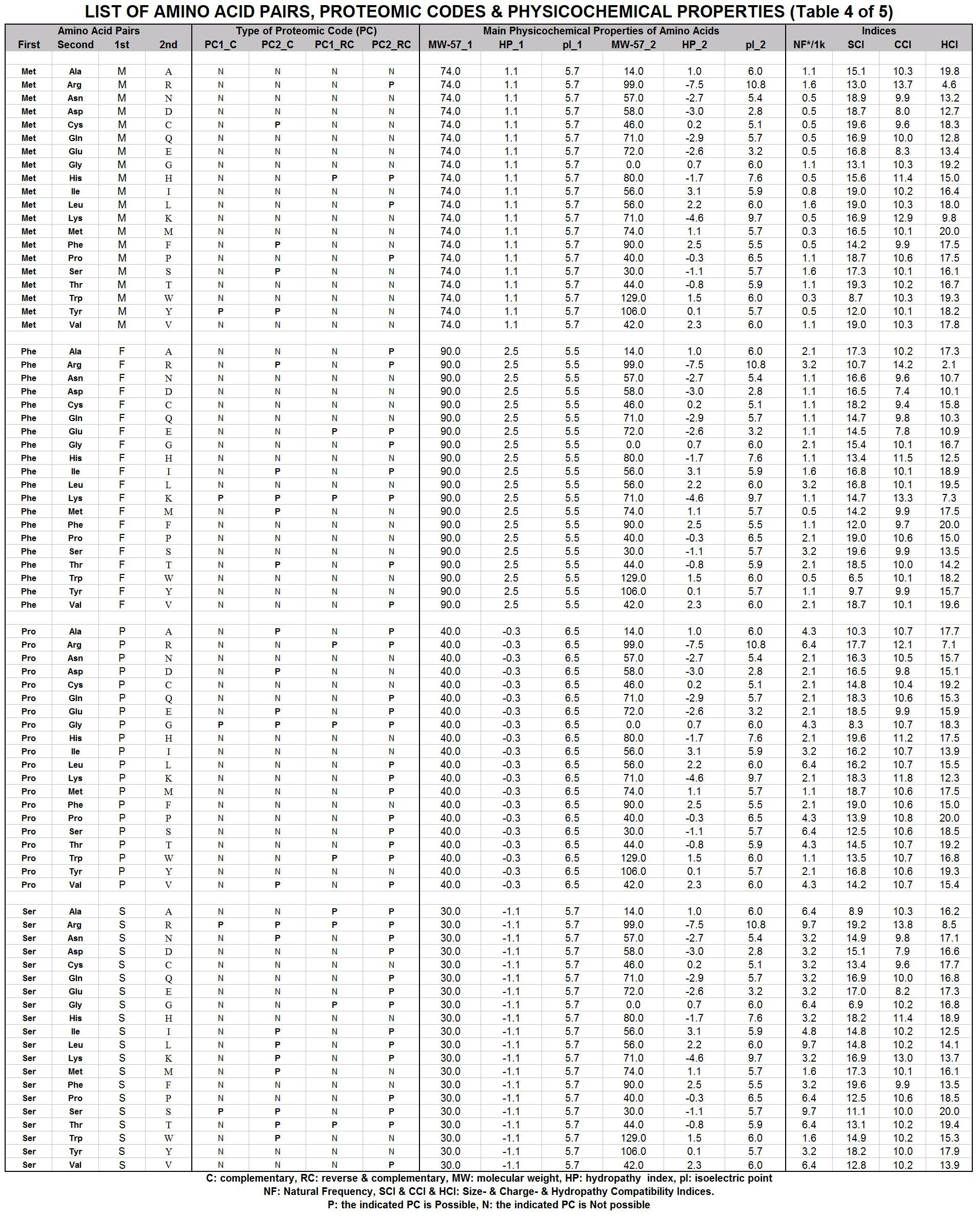


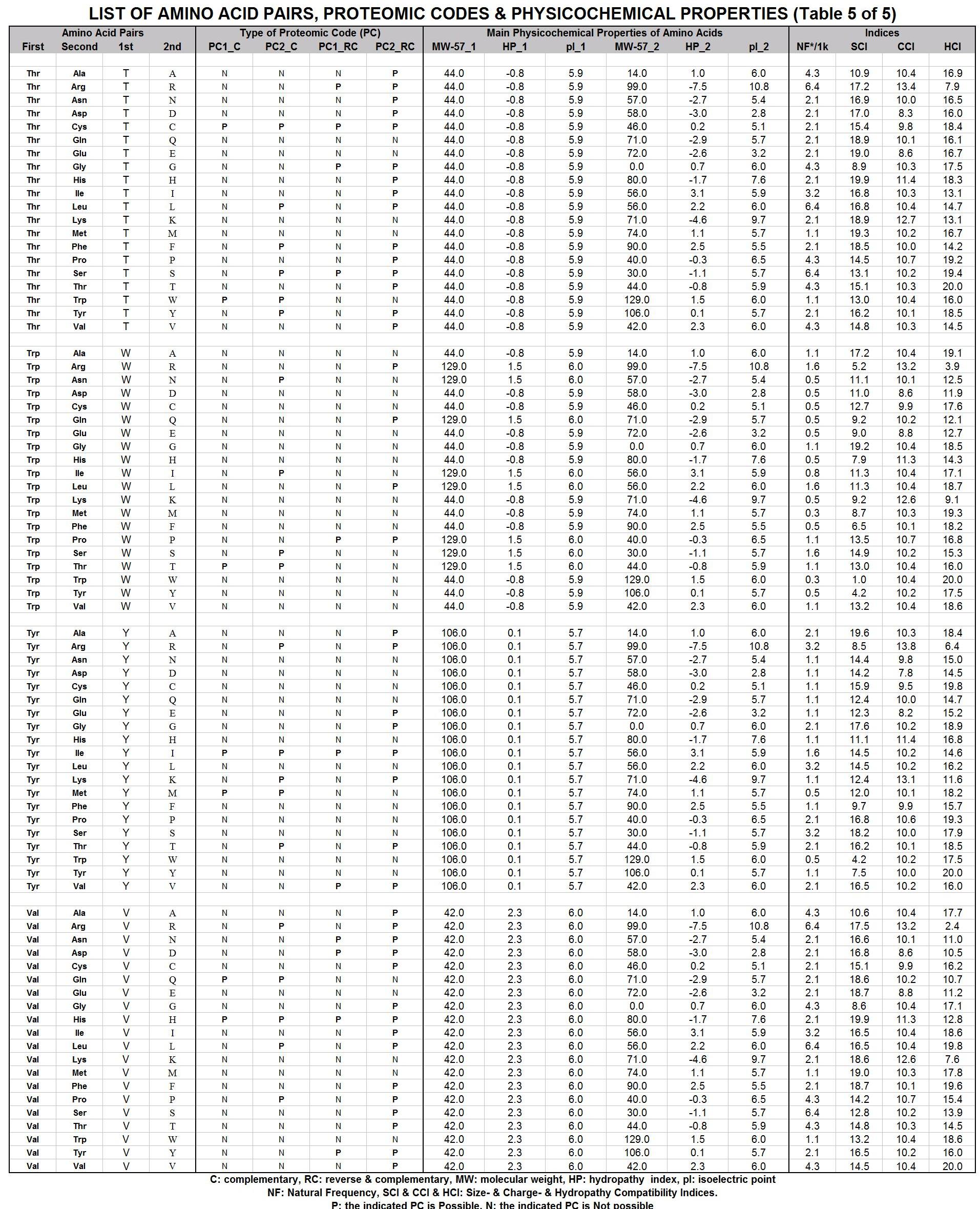

Supplement: Additional file 2 — List of Amino Acid Pairs, Proteomic Codes & Physicochemical Properties. List of Amino Acid Pairs, Proteomic Codes & Physicochemical Properties. [file 1742-4682-4-45-S2.doc]
